# Deep multimodal learning for domain-level cognitive decline prediction in Alzheimer's disease

**DOI:** 10.3389/frai.2025.1731062

**Published:** 2025-12-17

**Authors:** Fernando García-Gutiérrez, Jordi A. Matias-Guiu, José L. Ayala

**Affiliations:** 1 Department of Computer Architecture and Automation, Universidad Complutense de Madrid, Madrid, Spain; 2 Department of Neurology, Hospital Clínico San Carlos, Instituto de Investigación Sanitaria San Carlos (IdISSC), Madrid, Spain

**Keywords:** automated pattern recognition, artificial intelligence, machine learning, neuroimaging, neurodegenerative diseases, Alzheimer's disease

## Abstract

**Introduction:**

Alzheimer's disease (AD) is characterized by significant variability in clinical progression; however, few studies have focused on developing models to predict cognitive decline. Anticipating these trajectories is essential for patient management, care planning, and developing new treatments. This study explores the potential of artificial intelligence (AI) techniques to model neurocognitive trajectories from multimodal neuroimaging data and further investigates different data representation frameworks.

**Methods:**

Using information from 653 participants from the Alzheimer's Disease Neuroimaging Initiative (ADNI), we developed models to predict future clinical diagnoses and cognitive decline, both quantitatively (rate of decline) and qualitatively (presence or absence of decline). Input features included structural T1-weighted magnetic resonance imaging (MRI), [^18^F]-fluorodeoxyglucose positron emission tomography (FDG-PET), [^18^F]-florbetapir PET (AV45-PET), neuropsychological assessments, and demographic variables. Several information representation strategies were explored, including tabular data models, convolutional neural networks (CNNs), and graph neural networks (GNNs). Furthermore, to maximize the use of all available information, we proposed a modeling framework that performed modality-specific pre-training to learn feature embeddings, which were then integrated through a late-fusion layer to produce a unified representation for downstream prediction.

**Results:**

The modeling strategies demonstrated good predictive performance for future clinical diagnoses, consistent with previous studies (F1 = 0.779). Quantitative models explained approximately 29.4%–36.0% of the variance in cognitive decline. In the qualitative analysis, the models achieved AUC values above 0.83 when predicting cognitive deterioration in the memory, language, and executive function domains. Architecturally, CNN- and GNN-based models yielded the best performance, and the proposed pre-training strategy consistently improved predictive accuracy.

**Conclusions:**

This study demonstrates that AI techniques can capture patterns of cognitive decline by exploiting multimodal neuroimaging data. These findings contribute to the development of more precise phenotyping approaches for neurodegenerative patterns in AD.

## 1 Introduction

Alzheimer's disease (AD) represents the predominant cause of dementia worldwide (Alzheimer's Association, 2024). In its typical course, AD progresses gradually, beginning with a prolonged prodromal phase characterized by subtle memory impairments. As the disease advances, cognitive deficits become more pronounced and extend to other domains, eventually resulting in significant functional decline (Peña-Casanova et al., 2012).

Nevertheless, the progression of AD exhibits considerable interindividual variability. This heterogeneity is influenced by a combination of environment, genetic predisposition, and levels of cognitive reserve, among other factors (Hersi et al., 2017; Duara and Barker, 2022). Moreover, the presence of copathology further increases the variability in neurocognitive trajectories (Lam et al., 2013).

In this context, despite advancements in diagnostic and early detection methods (Ansart et al., 2021; Garćıa-Gutiérrez et al., 2022, 2024a; Sharma et al., 2023; Lee et al., 2024), tools for estimating cognitive decline remain underdeveloped. This limitation is particularly critical, as accurate trajectory prediction is essential for clinical decision-making, therapeutic planning, and patient selection for clinical trials (Abdelnour et al., 2022; Hampel et al., 2022). In fact, anticipating disease progression can substantially reduce the number of participants needed in clinical trials, with corresponding reductions in cost and patient exposure (Duara and Barker, 2022).

Accordingly, much of the existing research on modeling cognitive decline has adopted a qualitative approach, primarily focusing on transitions between clinical stages, such as from mild cognitive impairment (MCI) to dementia, with reported classification accuracies ranging from 75% to 95% (Ansart et al., 2021; Sharma et al., 2023; Garćıa-Gutiérrez et al., 2024b; Yang et al., 2025). However, predicting diagnostic transitions tends to oversimplify the complexity of cognitive decline, as it overlooks the finer-grained dynamics of domain-specific cognitive trajectories.

In contrast, a smaller subset of studies has aimed to model cognitive decline quantitatively. These efforts typically involve forecasting future scores on a limited number of neuropsychological assessments within defined time windows (Dansson et al., 2021; Lei et al., 2022; Maheux et al., 2023; Devanarayan et al., 2024). Commonly targeted measures include the Mini-Mental State Examination (MMSE), the Clinical Dementia Rating (CDR), and the cognitive sub-scale of the Alzheimer's Disease Assessment Scale (ADAS-Cog), often in the context of the TADPOLE challenge (Marinescu et al., 2019). Notably, recent studies have expanded the range of neuropsychological tests considered, incorporating measures such as the Rey Auditory Verbal Learning Test (RAVLT) (Seo et al., 2024), the Preclinical Alzheimer's Cognitive Composite (Devanarayan et al., 2025), and composite cognitive domains (Moradi et al., 2025). These advancements represent meaningful progress toward modeling cognitive decline with enhanced clinical relevance.

Furthermore, although these studies utilize a wide range of biomarkers for modeling, the representation of neuroimaging data remains restrictive. Typically, features are extracted from a single imaging modality, or occasionally from multiple modalities, and treated as tabular data. However, this ignores spatial and topological information inherent to brain structure (Maheux et al., 2023; Devanarayan et al., 2024).

This approach contrasts with other applications in which the superiority of artificial intelligence (AI) models incorporating three-dimensional or graph-based information has been well established (Li et al., 2021; Sharma et al., 2023). This is particularly relevant given the inherent complexity of modeling cognitive decline, a task considerably more challenging than diagnosis or conversion prediction. Enhancing predictive performance in this setting requires more effective utilization of neuroimaging data, one of the most informative biomarkers (Nordberg et al., 2010).

In this context, we aim to model domain-specific cognitive trajectories and to predict diagnostic transitions between clinical stages of the AD continuum. To this end, we introduce a multimodal learning framework that exploits complementary data representations and maximizes the use of available neuroimaging information.

Whereas many prior approaches rely primarily on global cognitive assessments such as the MMSE, our method specifically targets the core cognitive domains typically affected in AD, including memory, language, visuospatial abilities, and executive functions. Furthermore, to obtain a more robust quantification of cognitive decline and to mitigate the noise associated with single time-point measurements, we focus on modeling longitudinal trajectories of cognitive performance.

Methodologically, the proposed framework incorporates two key strategies to improve predictive performance. First, we introduce a pre-training stage to exploit the full dataset, thereby alleviating common limitations associated with high-dimensional neuroimaging data and relatively small sample sizes. Second, we systematically explore alternative data representations to enhance model accuracy and efficiency. Specifically, we evaluate tabular features derived from multiple neuroimaging modalities, leverage three-dimensional information through convolutional neural networks (CNNs), and incorporate brain connectivity patterns via graph neural networks (GNNs).

Through this approach, we aim not only to enhance the predictive accuracy of cognitive decline but also to advance toward more detailed and clinically relevant modeling. This approach has the potential to optimize intervention strategies, support treatment personalization, and improve patient selection for clinical trials.

## 2 Methodology

### 2.1 Study cohort

This study used data from the Alzheimer's Disease Neuroimaging Initiative (ADNI) accessed on April 28, 2024 (https://adni.loni.usc.edu). Initiated in 2003, ADNI represents a collaborative effort aimed at exploring whether a combination of serial magnetic resonance imaging (MRI) and positron emission tomography (PET) scans, along with other biomarkers and clinical/neuropsychological evaluations, can effectively track the progression of MCI and early AD.

Models were developed to predict future diagnoses at two and four years, as well as cognitive decline (as defined in Section 2.4). The dataset used to model cognitive decline included 653 subjects. In contrast, for predicting future diagnoses, not all participants had diagnostic information available within a window of at least six months relative to each prediction point. Consequently, 519 and 354 subjects were included in the two- and four-year diagnosis prediction tasks, respectively.

Selection criteria included the availability of neuropsychological assessments, MRI, [^18^F]-fluorodeoxyglucose PET (FDG), and [^18^F]-florbetapir (AV45) PET data acquired within a three-month window, as well as a minimum follow-up period of two years from the initial neuropsychological visit. Additionally, since dementia patients already exhibit severe cognitive impairment, only individuals clinically diagnosed as cognitively normal (CN) or as having MCI at baseline were included. The socio-demographic characteristics of the sample are presented in Table 1.

**Table 1:** Clinical and socio-demographic variables of the sample used for modeling cognitive decline.

**Diagnosis**	**CN**	**MCI**
Sample size (N, %)	267 (40.9)	386 (59.1)
Age (Mean, SD)	74.4 (6.3)	72.3 (7.4)
Sex (% Female)	51.7	40.9
Years of formal education (Mean, SD)	16.8 (2.4)	16.2 (2.7)
MMSE (Mean, SD)	29.0 (1.2)	27.9 (1.8)
Years follow-up (Mean, SD)	6.6 (3.6)	5.3 (3.2)

CN, cognitively normal; MCI, mild cognitive impairment; MMSE, Mini-Mental State Examination; SD, standard deviation.

Moreover, the modeling framework presented in Section 2.5 includes a modality-specific pre-training step. For this pre-training, data from 1,668 subjects with associated neuroimaging information—diagnosed as CN, MCI, or dementia—were used.

### 2.2 Neuroimaging data

Raw high-resolution (1.5T and 3T) T1-weighted MRI magnetization-prepared rapid gradient echo (MPRAGE), FDG, and AV45 scans were processed. MRI and PET images were paired if acquired within three months of the corresponding MRI scan.

Firstly, brain extraction was performed on the MRI scans using *SynthStrip* (Hoopes et al., 2022), and bias field inhomogeneity was corrected using the N4 algorithm from Advanced Normalization Tools (ANTs) (Tustison et al., 2010). Then, gray matter (GM) and white matter (WM) were segmented using the *New Segment* tool in Statistical Parametric Mapping 12 (SPM12), implemented in MATLAB R2023b (MathWorks Inc.). Since bias correction had already been performed with ANTs, no additional bias regularization was applied in *New Segment*. Segmentation was performed using the default SPM12 tissue probability maps, with all other parameters left unchanged.

Separately, *dynamic* PET acquisitions were converted to *static* images following ADNI preprocessing protocols (Jagust et al., 2015). Static PET images were first pre-aligned to their corresponding MRI scans using FSL, applying a rigid-body transformation with normalized mutual information as the cost function. The PET images were then co-registered to the MRI scans using the *Co-register* function in SPM12, again using normalized mutual information as the cost function and applying a 3rd-degree spline interpolation with all other parameters set to default.

Thereafter, MRI scans were normalized using SPM12 to the MNI152 template (isotropic 1 mm^3^ voxel size) using a 7th-degree spline interpolation. The deformation fields obtained during normalization were applied to the PET acquisitions, along with GM and WM masks, transforming all images into the same reference space.

Afterwards, spatial smoothing with a full width at half maximum (FWHM) of 4 mm was performed using an isotropic Gaussian kernel. Additionally, for PET images, standardized uptake value ratios (SUVRs) were calculated using the whole cerebellum as the reference region (Dukart et al., 2010; Shekari et al., 2024).

Finally, outlier detection methods were applied to the processed images (see Appendix 1), followed by manual inspection to identify potential errors in the preprocessing pipeline. Cases exhibiting acquisition-related artifacts or processing failures were either reprocessed or excluded from the dataset. The final dataset used for modeling (Section 2.5) consisted of 3,861 MRI scans, 2,548 FDG scans, and 1,403 AV45 scans.

### 2.3 Neuropsychological data

Neurocognitive performance was evaluated using neuropsychological composite scores covering four cognitive domains: memory, language, executive function, and visuospatial abilities. These composites were derived via structural equation modeling, following established methodologies (Park et al., 2012; Gibbons et al., 2012; Crane et al., 2012; Choi et al., 2020).

For the memory domain, subscores from the RAVLT (trials 1, 2, and 6, 30-min delayed recall, and recognition) were included, along with word recall, delayed recall, and recognition sub-scores from the ADAS-Cog (Rey, 1958; Mohs et al., 1997). Language ability was assessed using the Category Fluency Test (Morris et al., 1989), the Naming Objects and Fingers item, and word-finding difficulty ratings from the ADAS-Cog, as well as the Boston Naming Test (BNT) (Kaplan et al., 2001). Executive function was characterized using scores from the Trail Making Test (log-transformed completion time in seconds) (Reitan and Wolfson, 2014) and the ADAS-Cog attention subscore (number cancellation). Visuospatial abilities were evaluated using the total score from the Clock Drawing Test (copy and draw) (Goodglass et al., 2001), and the constructive and ideational praxis subscores from the ADAS-Cog.

Missing data (< 4%, except for BNT) were imputed using multivariate imputation by chained equations (White et al., 2011), employing a Random Forest (RF) as a surrogate model. Given that the BNT total score was only available for ADNI 1/GO/2 cohorts, this resulted in 20.4% missingness (16.5% in the longitudinal subset), and a separate imputation strategy was employed. In this case, missing values were imputed using a RF model, incorporating the remaining neuropsychological tests, age, sex, and years of education as predictor variables. Experimental validation (Appendix 2) showed a correlation exceeding 0.75 with the observed values and a mean absolute error of 2.2 for this approach.

Composite scores were estimated by fixing the latent factor variance to one for model identification (Hair et al., 2021). The executive function, language, and visuospatial composites were computed using the weighted least squares mean and variance adjusted estimator, treating the ADAS-Cog attention number cancellation, the language-related items, and visuospatial sub-scores as ordinal categorical variables. For the memory composite, the robust maximum likelihood estimator was used.

To enhance interpretability, the resulting composite scores were scaled according to Equation 1, where a value of 0 corresponded to the median of individuals with mild dementia ($P_{50}^{dem}$), and a value of 100 corresponded to the median of CN subjects ($P_{50}^{cn}$):


$$\begin{array}{l} x^{\text{adj}}=\left(\frac{x-P_{50}^{\;\text{dem}}}{P_{50}^{\;\text{cn}}-P_{50}^{\;\text{dem}}}\right)\cdot 100 \end{array}$$ (1)

Lastly, all composite scores were adjusted for age and educational level using normative data. Normative subjects were defined as those with: a CN diagnosis sustained across all visits, a CDR of 0.0, a MMSE score greater than 26, and negative amyloid status based on the SUVR threshold defined in Johnson et al. (2013).

Data imputation was performed using Python (v3.11.5) and the *Scikit-Learn* (v1.6.1) library (Pedregosa et al., 2011), while composite score calculations were conducted in R (v4.4.2) with the *lavaan* (v0.6-19) package (Rosseel, 2012). For additional details on composite score definitions, computations, and analysis, refer to Appendix 3.

### 2.4 Definition of cognitive decline

This study aimed to develop models for predicting cognitive decline across the cognitive domains described in Section 2.3. Cognitive decline was defined using two complementary criteria: (1) a quantitative measure, reflecting the annual rate of cognitive function deterioration, and (2) a qualitative measure, determined as the likelihood of an individual experiencing significant cognitive decline over time. These definitions were designed to reduce the variability inherent in single-time-point assessments of cognitive performance, by incorporating more robust longitudinal information on neurocognitive trajectories.

Quantitative cognitive decline was defined based on the slope of cognitive performance over time, measured from the neuroimaging acquisition used as model input (Franzmeier et al., 2020). Alternatively, the binary classification of cognitive decline (*stable* vs. *decliner*) was determined using domain-specific cut-off points. These thresholds were defined based on the 5th percentile of cognitive decline observed in individuals with a clinical diagnosis of CN and sustained amyloid-negative status over time. Accordingly, an individual was classified as exhibiting abnormal cognitive decline if the individual's rate of decline fell below that of 95% of control subjects.

Supplementary Table A3 summarizes the statistics of cognitive decline and their relationship to diagnostic changes.

### 2.5 Machine learning modeling

**Figure 1:**
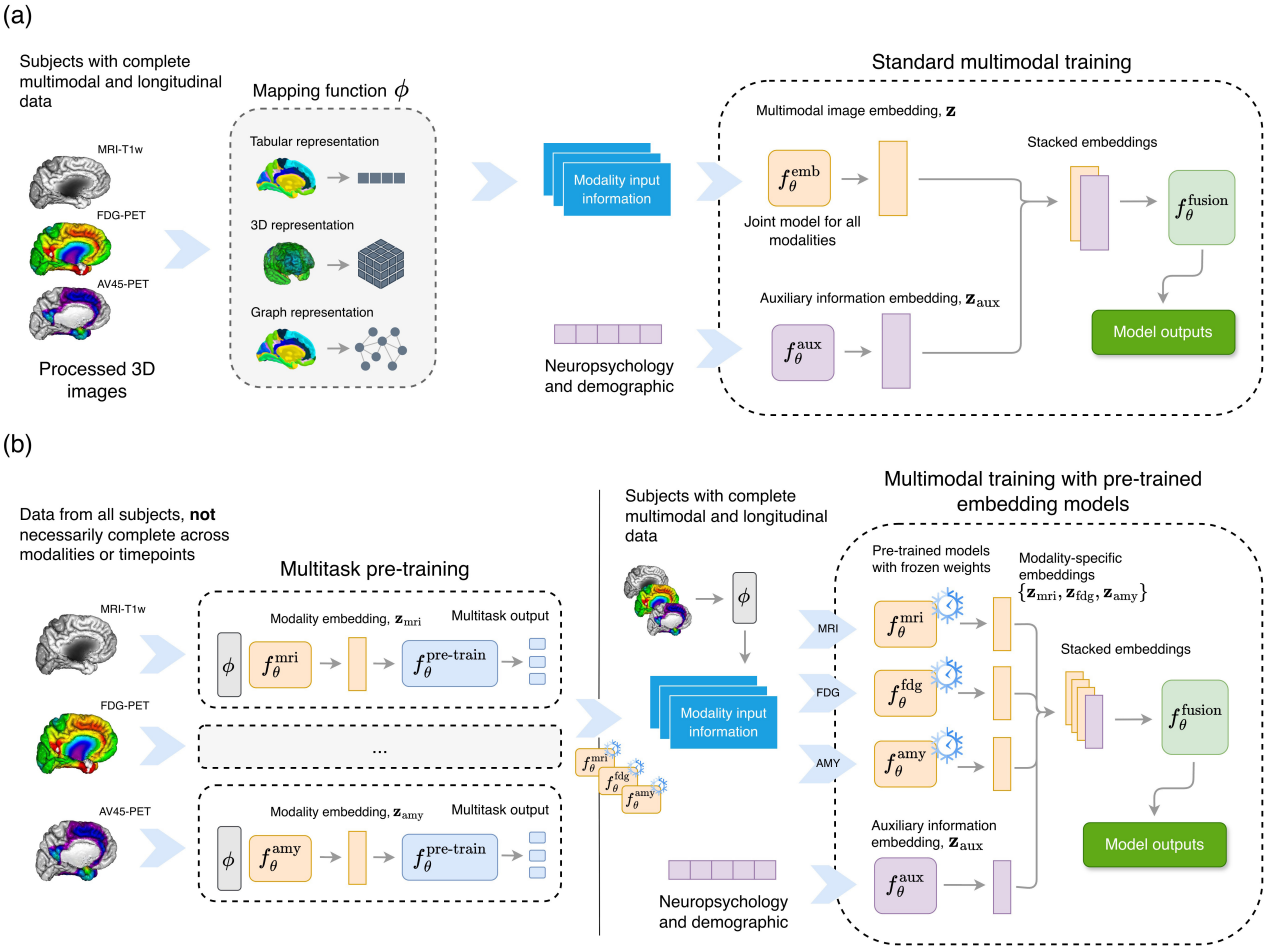
Overview of the multimodal learning framework. **(a)** *Standard multimodal training*. Processed 3D neuroimaging data from all modalities (MRI, FDG-PET, and AV45-PET) are first mapped into structured representations through the mapping function ϕ (e.g., tabular, 3D, or graph-based formats), and subsequently processed by a joint image-embedding model. In parallel, demographic and neuropsychological variables are encoded by an auxiliary embedding model. The resulting imaging and auxiliary embeddings are then concatenated and passed to a fusion network to produce the final outputs. **(b)** *Multimodal training with pre-trained embedding models*. Each imaging modality undergoes an independent multitask pre-training phase to learn modality-specific embedding models using all available data, including subjects with incomplete modalities or longitudinal follow-up data. The pre-trained modality-specific models are then frozen and used to generate fixed modality-specific embeddings which, together with embeddings derived from demographic and neuropsychological information, are concatenated and fed into a multimodal fusion network to generate the final outputs.

Figure 1 provides an overview of the modeling framework. The following subsections describe the different strategies used for modeling. These include the use of multimodal models (Section 2.5.1), as commonly developed in the literature (Franzmeier et al., 2020; Nguyen et al., 2020; Gravina et al., 2024), as well as the main contribution of this paper, which incorporates a pre-training step and late fusion to maximize the use of the available information (Section 2.5.2). Finally, the different parameterizations used to exploit the neuroimaging data are described in Sections 2.5.3–2.5.5.

#### 2.5.1 Multimodal data modeling

The primary objective of this study was to approximate each target variable, *y*, from a set of three-dimensional images, $\text{X}\in {\mathbb{R}}^{M\times d_{x}\times d_{y}\times d_{z}}$, derived from *M* modalities. In general terms, the goal was to approximate:


$$\begin{array}{l} y\approx f_{\theta }\left(\phi \left(\text{X}\right)\right), \end{array}$$ (2)

where ϕ represents a mapping function, and θ denotes the learnable parameters of the predictive model *f*. In this context, the role of ϕ was to adapt the three-dimensional data to match the expected input dimensions of *f*_θ_. Furthermore, depending on the nature of each target variable *y*, continuous or categorical, models were trained to perform either regression or classification tasks.

In addition, to incorporate auxiliary information into the model, such as demographic or neuropsychological data, an information fusion layer was introduced. In this case, given tabular input data ${\text{x}}_{\text{aux}}\in {\mathbb{R}}^{t}$, an auxiliary model $f_{\theta }^{\text{aux}}$ was defined, and the original formulation *f*_θ_ was partitioned into an output model $f_{\theta }^{\text{fusion}}$, and an embedding model, $f_{\theta }^{\text{emb}}$, incorporating a multi-head attention mechanism (MHA) (Vaswani et al., 2017) to fuse the information (Figure 1a):


$$\begin{array}{l} y\approx f_{\theta }^{\text{fusion}}\left(\text{MHA}\left(\mathrm{stack}\left[f_{\theta }^{\text{emb}}\left(\phi \left(\text{X}\right)\right),f_{\theta }^{\text{aux}}\left({\text{x}}_{\text{aux}}\right)\right]\right)\right). \end{array}$$ (3)

#### 2.5.2 Modeling with pre-training

##### 2.5.2.1 Pre-training strategy

Since not all subjects had data available for every modality or sufficient longitudinal follow-up with neuropsychological assessments, an alternative formulation to Equation 2, incorporating a pre-training step, was introduced. The goal of this strategy was to maximize the use of all available information.

At this stage, each modality *m* was considered independently, and a multitask model was trained to estimate a set of *d* target variables related to the main study objectives, denoted by **y**′ and approximated as follows:


$$\begin{array}{l} {\text{y}}^{\prime }\approx f_{\theta }^{\text{pre-train}}\left(f_{\theta }^{m}\left(\phi^{m}\left({\text{X}}_{m}\right)\right)\right). \end{array}$$ (4)

Here, $f_{\theta }^{\text{pre-train}}$ denotes a model used exclusively during the pre-training phase, based on a shallow feed-forward network (FFN) with multitask projection heads. Meanwhile, $f_{\theta }^{m}$ represents the modality-specific component of the model that undergoes pre-training. In this formulation, $f_{\theta }^{m}$ is responsible for learning the representation associated with modality *m*, with ϕ^*m*^ denoting its corresponding mapping function.

Therefore, the pre-training loss function was formulated as follows:


$$\begin{array}{l} L^{\text{pre-train}}\left({\text{y}}^{\prime },{\hat{y}}^{\prime }\right)=\;\overset{B}{\underset{b}{\sum }}w_{b}\cdot L^{\text{BCE}}\left(y_{b}^{\prime },{\hat{y}}_{b}^{\prime }\right)\\ \;+\overset{C}{\underset{c}{\sum }}w_{c}\cdot L^{\text{CE}}\left({\text{y}}_{c}^{\prime },{{\hat{y}}^{\prime }}_{c}\right)\\ \;+\overset{R}{\underset{r}{\sum }}w_{r}\cdot L^{\text{Huber}}\left(y_{r}^{\prime },{\hat{y}}_{r}^{\prime }\right), \end{array}$$ (5)

where ℒ^BCE^ denotes the binary cross-entropy loss computed over the ℬ binary classification tasks; ℒ^CE^ corresponds to the categorical cross-entropy loss calculated for the 𝒞 multiclass classification problems; and ℒ^Huber^ refers to the Huber loss used for the ℛ regression tasks. The vector **w** contains the weights assigned to each task in **y**′. Moreover, to handle missing values, a mask was applied to the entries of the predicted output vector ŷ′, ignoring target variables for which ground truth data were unavailable for a given subject.

##### 2.5.2.2 Fusion model for multimodal integration

After pre-training, the learned embedding representations—defined as ${\text{z}}_{m}=f_{\theta }^{m}\left({\text{X}}_{m}\right)$—from each imaging modality were integrated using a fusion model:


$$\begin{array}{l} y\approx f_{\theta }^{\text{fusion}}\left(\text{MHA}\left(\mathrm{stack}\left[{\text{W}}_{\text{mri}}{\text{ z}}_{\text{mri}},\;{\text{W}}_{\text{fdg}}{\text{ z}}_{\text{fdg}},\;{\text{W}}_{\text{amy}}{\text{ z}}_{\text{amy}}\right]\right)\right), \end{array}$$ (6)

where **W**_*m*_ represented linear projections that mapped the embeddings to a common latent space of dimension *D*; and $f_{\theta }^{\text{fusion}}$ was the fine-tuned model for each of the addressed objectives. Specifically, $f_{\theta }^{\text{fusion}}$ consisted of a FFN, which incorporated either a softmax activation function for multiclass classification tasks or linear projections for regression tasks.

Similar to Equation 3, demographic and neuropsychological data were optionally integrated into the fusion models. However, due to the low dimensionality of these features, they were not considered during the pre-training phase. Instead, the information was processed through a simple FFN and subsequently incorporated into the fusion model (Equation 6), stacked with the rest of the embedding projections (Figure 1b).

#### 2.5.3 Feed-forward network based models

Feed-forward network-based models represent the simplest neural network architecture. These models apply a sequence of linear transformations followed by nonlinear activation functions, optionally incorporating dropout and normalization layers to stabilize training.

Following the framework presented in previous sections, the mapping function ϕ was designed to adapt the three-dimensional data to the FFNs. This function was defined to aggregate the information into regions of interest (ROIs) using the Automated Anatomical Labeling (AAL) brain atlas (Rolls et al., 2020). Specifically, for each ROI, the ϕ function calculated the GM and WM volumes for the MRI data ($\phi^{\text{mri}}:{\mathbb{R}}^{d_{x}\times d_{y}\times d_{z}}\to {\mathbb{R}}^{232}$) and computed the mean and standard deviation for the SUVR values of the PET data ($\phi^{\left\{\text{fdg},\text{amy}\right\}}:{\mathbb{R}}^{d_{x}\times d_{y}\times d_{z}}\to {\mathbb{R}}^{232}$).

In this parametrization, the models $f_{\theta }^{\text{emb}}$ and $f_{\theta }^{m}$ were implemented as stacks of multiple layers. For $f_{\theta }^{\text{emb}}$, information from the different modalities was concatenated into a single vector. During experimentation, various hyperparameters were explored, including the number and width of the layers, the activation functions used, dropout rates, and the application of batch normalization. Details of the hyperparameter configurations are provided in Appendix 4.

#### 2.5.4 3D convolutional network based models

Three-dimensional CNNs extend classical CNNs, originally designed for the two-dimensional imaging domain, by accommodating an additional dimension. The core idea behind these models is the use of convolution and pooling operations, which enable parameter sharing and the generation of equivariant representations. These properties make 3D-CNNs efficient and robust for processing high-dimensional neuroimaging data (Spasov et al., 2019; Peng et al., 2021; Gravina et al., 2024).

In this approach, to reduce the memory requirements associated with the models, the ϕ functions were implemented by downsampling the image dimensions by a factor of 2 using a continuous interpolation. Additionally, to mitigate the heterogeneity of the MRI scans, the ϕ^mri^ function performed image normalization by dividing the intensity values by the median WM value.

For the model architectures evaluated for $f_{\theta }^{\text{emb}}$ and $f_{\theta }^{m}$, two distinct parameterizations were considered. The first followed the scaffold proposed in Garćıa-Gutiérrez et al. (2024b), while the second was based on a DenseNet architecture (Huang et al., 2017) adapted for processing 3D data. In the specific case of $f_{\theta }^{\text{emb}}$ (model without pre-training, Equation 3), images from different modalities were provided as different input channels. During the experiments, various architectural configurations, normalization layers, and dropout rates were explored.

#### 2.5.5 Graph neural network based models

Unlike the models from previous sections applied to data within a Euclidean domain, models based on GNNs operate on graphs. Formally, a graph 𝒢(𝒱, ℰ) is defined as a set of 𝒱 nodes containing *d* node features, **X**∈ℝ^|𝒱| × *d*^, connected by a set of ℰ edges. The aim of the GNNs is to generate a series of node embeddings **h**_*u*_∀*u*∈𝒱 by applying the following operations over *k* message-passing layers:


$$\begin{array}{l} {\text{h}}_{u}^{\left(k+1\right)}={\text{UPDATE}}^{\left(k\right)}\left({\text{h}}_{u}^{\left(k\right)},\;\text{AGGREGATE }\left(\left\{{\text{h}}_{v}^{\left(k\right)}\;\forall v\in N\left(u\right)\right\}\right)\right) \end{array}$$ (7)

where UPDATE and AGGREGATE are differentiable functions that determine how the information is aggregated and updated, 𝒩(*u*) represents the neighborhood of node *u*, and ${\text{h}}_{u}^{\left(0\right)}=X_{u}$. Thus, in each iteration, each node aggregates information from its neighborhood, capturing both the structural properties of 𝒢 and the features of neighboring nodes (Corso et al., 2024).

In this context, to define the input graphs of the models, the graphical lasso method was used (Huang et al., 2010). Briefly, this method estimates the inverse covariance matrix by applying *L*1 regularization, weighted by λ (Friedman et al., 2008). To fit the model, FDG data from all CN and MCI participants were used. As in Section 2.5.3, brain metabolism data were aggregated into ROIs using the AAL atlas, and the mean SUVR values were used as model input. The estimation resulted in an undirected unweighted connectivity graph with 116 nodes, exhibiting a specific pattern of connections regulated by λ. In the experiments, λ was one of the hyperparameters explored.

Accordingly, the mapping function ϕ was defined to map the three-dimensional image information onto a graph domain. Specifically, the ϕ^mri^ function used GM and WM values as node features, while the ϕ^{fdg, amy}^ functions incorporated the mean and standard deviation of the SUVR values.

The models $f_{\theta }^{\text{emb}}$ and $f_{\theta }^{m}$ were implemented according to the formulation of Equation 7. In the $f_{\theta }^{\text{emb}}$ model, features from different neuroimaging modalities were concatenated into a single vector and treated as node features. At the architectural level, different types of aggregation and update layers were explored, including Graph Convolutional Networks (GCNs) with symmetric normalization (Kipf and Welling, 2016); Graph Attention Networks (GATs) (Veličković et al., 2017), which included self-loops and followed the attention mechanism of Bahdanau et al. (2014); and Graph Isomorphism Networks (GINs) (Xu et al., 2018a).

The explored model hyperparameters included the use of jumping knowledge connections (Xu et al., 2018b), mean and sum graph pooling operators, the number of message-passing layers, and the size of the embedding representations. Furthermore, all models incorporated graph normalization (Cai et al., 2021), applied a dropout rate of 0.1, and used ELU as the activation function. For more details on the implementation of the models, see Appendix 4.

### 2.6 Experimental setup

#### 2.6.1 Model evaluation

To evaluate the models, the data were split into training (60%), validation (10%), and test sets (30%). The training set was used for model fitting, the validation set for hyperparameter tuning and model selection, and the test set for final evaluation. For model pre-training, the data were divided into training (85%) and validation (15%), ensuring that no test data were used in this step.

To evaluate the results and select the models, the Matthews Correlation Coefficient (MCC) (Chicco and Jurman, 2023) was used as the primary metric for classification tasks, and the explained variance (EV) for regression tasks. For the final evaluation, additional metrics were also considered, including the F1-score (weighted-macro F1-score for multiclass problems) and the area under the curve (AUC) for classification, and Pearson's correlation coefficient for regression.

#### 2.6.2 Optimization and implementation details

Section 2.5 described the different parameterizations used for $f_{\theta }^{\text{emb}}$ and $f_{\theta }^{m}$, which were subjected to hyperparameter optimization as outlined in Appendix 4. Implementation details of the components $f_{\theta }^{\text{fusion}}$, $f_{\theta }^{\text{aux}}$, $f_{\theta }^{\text{pre-train}}$ and MHA (Equations 3, 6), along with aspects related to model fitting, are provided below:

$f_{\theta }^{\text{fusion}}$: this component of the model consisted of a four-layer FFN with hidden layer dimensions of 64, 48, 32, and 16, respectively. SiLU activation functions were used (Elfwing et al., 2018), and the activation function of the output layer was adapted based on the specific task. Batch normalization was applied to the first four layers, and a dropout rate of 0.1 was used in the first layer.

$f_{\theta }^{\text{aux}}$: this module was implemented as a two-layer FFN with a hidden dimension of 32, using SiLU activation functions. A dropout rate of 0.1 was applied to the first layer, and batch normalization was incorporated in both layers. Additionally, a linear projection was included to align the output dimensionality with that required by the embeddings in Equations 3, 6.

$f_{\theta }^{\text{pre-train}}$: this component was designed as a multitask adapter to support the pre-training stage of $f_{\theta }^{m}$ (Equation 4). Its architecture was similar to $f_{\theta }^{\text{fusion}}$, with the exception that the final two layers, with dimensions 32 and 16, were replicated *d* times to serve as task-specific heads.

*Multi-head attention* (MHA): the attention component of Equations 3, 6 consisted of an 8-head MHA layer with an embedding dimension of 16. A dropout rate of 0.1 was applied, and the resulting attention-weighted embeddings were aggregated via mean pooling.

All models were trained using backpropagation and optimized with the Adam algorithm (Kingma and Ba, 2014). The learning rate, batch size, and number of training epochs were treated as tunable hyperparameters. A learning rate scheduler was applied to all models, reducing the learning rate by a factor of 0.5 every 20% of the total number of epochs. Early stopping was also employed, terminating training when the validation loss failed to improve for a number of consecutive epochs corresponding to 10% of the total training duration.

For simplicity, in Equation 5, all task weights were set to 1, and the Huber loss function was used with a fixed δ value of 2.5. During pre-training, the predicted targets included the current diagnosis, the diagnosis at two and four years, and quantitative measures of cognitive decline.

Moreover, for modeling quantitative cognitive decline, the target variables were standardized to z-scores using the mean and standard deviation of the training set. Prior to standardization, outliers were trimmed based on the 5th and 95th percentiles. Similarly, standardization (without trimming) was applied to the input data for all models. In the models that included neuropsychological and demographic information, the tests considered as input were those described in Section 2.3, and the demographic variables were age, sex, and educational level.

All developed models were compared with RF baselines trained with (i) neuropsychological and demographic data only, (ii) neuroimaging data only, and (iii) a combination of neuropsychological, demographic, and neuroimaging data.

Experiments were performed using an NVIDIA RTX A5000 (CUDA 12.6). The models were developed in Python (v3.11.5), using the PyTorch (v2.5.1) and PyTorch Geometric (v2.5.1) libraries. The code used for data preparation and model implementation is provided on GitHub. For more details on the hyperparameters explored for each type of model, see Appendix 4.

## 3 Results

The following sections present the results of future diagnosis and cognitive decline prediction. Models were labeled as *ni* (non-imaging) when neuroimaging data were excluded, *nd* when neuropsychological and demographic information were included, and *pt* when pre-training was incorporated. By default, if none of these labels were specified, it was assumed that the models had been trained using only neuroimaging data.

### 3.1 Diagnosis prediction at 2 years

**Figure 2:**
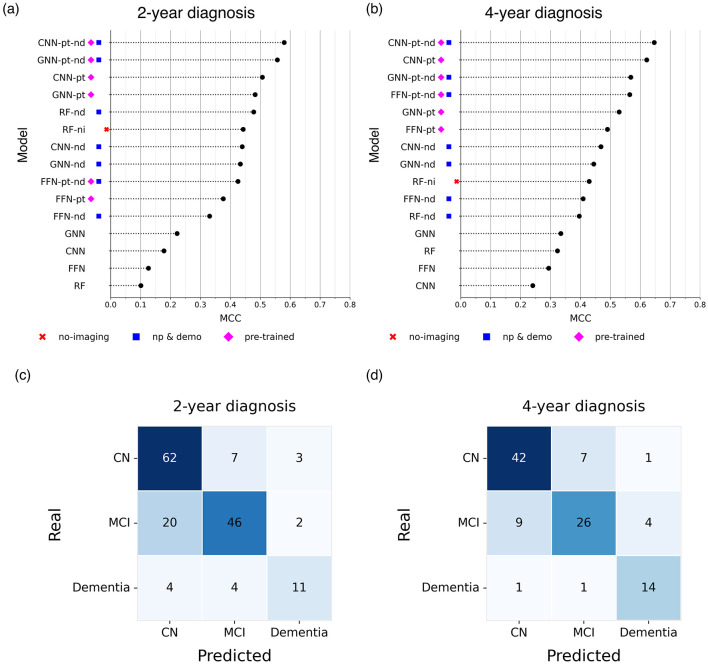
Comparison of the Matthews Correlation Coefficient (MCC) of the best models **(a, b)** and confusion matrices associated with the best model **(c, d)** for predicting future diagnoses at **(a, c)** 2 years and **(b, d)** 4 years. Higher MCC values indicate better predictive performance. The figure presents results from the best-performing models obtained by excluding neuroimaging data (no-imaging), incorporating neuropsychological and demographic information (np & demo), and including a pre-training step (pre-trained).

In the two-year diagnostic prediction task, the best performance was achieved by models that incorporated both neuropsychological and demographic information, as well as a pre-training phase (Figure 2a). Notably, CNN- and GNN-based architectures outperformed all other models. The CNN-pt-nd model yielded the highest performance (MCC 0.580, F1 0.745), followed closely by the GNN-pt-nd model (MCC 0.557, F1 0.736) (Figure 2c).

In contrast, the baseline RF model trained exclusively on neuroimaging data performed the worst (MCC 0.101, F1 0.460). However, performance improved markedly when neuropsychological and demographic features were included, either alone or in combination with neuroimaging data (MCC 0.478, F1 0.677).

Similarly, FFNs, CNNs, and GNNs trained solely on neuroimaging data without pre-training achieved modest results (e.g., GNN: MCC 0.222, F1 0.544), although they still outperformed the neuroimaging-only RF baseline. When neuropsychological and demographic features were added to these models, performance improved substantially (e.g., CNN-nd: MCC 0.439, F1 0.665), reaching levels comparable to the RF-nd model.

### 3.2 Diagnosis prediction at 4 years

In the four-year diagnostic prediction task, models consistently exhibited greater predictive capacity compared to the 2-year results (Figure 2b).

Within the baseline models, the best performance was achieved by the RF-ni model (MCC 0.429, F1 0.653), while those relying solely on neuroimaging data without pre-training showed the weakest performance (MCC < 0.4). In contrast, CNN and GNN models incorporating neuropsychological and demographic information outperformed all RF-based baselines, with the CNN-nd model achieving an MCC of 0.468 and F1 of 0.672.

Across all configurations, pre-trained models consistently outperformed models without pre-training. Notably, CNNs demonstrated the highest predictive accuracy, with the CNN-pt-nd model achieving the highest performance (MCC 0.646, F1 0.779) (Figure 2d).

### 3.3 Detection of diagnosis transitions

Predictive models for dementia conversion are widely explored in the literature (Ansart et al., 2021; Sharma et al., 2023; Garćıa-Gutiérrez et al., 2024b; Lee et al., 2024), typically aiming to distinguish individuals with MCI who progress to dementia (pMCI) from those who remain stable (sMCI).

In contrast, this study directly modeled future clinical diagnoses without explicitly targeting conversion events. Nevertheless, the model outputs enabled retrospective assessment of the models' ability to identify dementia conversion and other diagnostic transitions.

In this context, the best-performing model (CNN-pt-nd) achieved an AUC of 0.772 and an F1 of 0.629 for detecting sMCI/pMCI at two years, and an AUC of 0.909 with an F1 of 0.800 at 4 years.

Similarly, for identifying diagnostic transitions over a maximum period of 4 years—such as transitions from CN to MCI or dementia, and from MCI to dementia—the best model reached an AUC of 0.795 and an F1 of 0.645.

Appendix 6 shows the results obtained for the different models explored in the study.

### 3.4 Quantitative prediction of cognitive decline

Table 2 summarizes the performance of the evaluated models in predicting continuous cognitive decline (see Section 2.4). Additional metrics are detailed in Appendix 6.

**Table 2:** Explained variance of regression models predicting continuous measures of cognitive decline.

**Model**	**Memory**	**Language**	**Visuospatial**	**Executive**
RF-ni	0.06 [–0.04, 0.14]	0.20 [0.08, 0.30]	0.26 [0.16, 0.35]	0.12 [–0.05, 0.26]
RF	0.17 [0.08, 0.26]	0.11 [–0.00, 0.21]	0.10 [–0.00, 0.19]	0.20 [0.08, 0.29]
FFN	0.07 [–0.06, 0.19]	0.18 [0.02, 0.32]	0.06 [–0.08, 0.17]	0.21 [0.09, 0.33]
CNN	0.11 [0.03, 0.18]	0.09 [0.01, 0.17]	0.07 [–0.03, 0.16]	0.12 [–0.02, 0.22]
GNN	0.19 [0.09, 0.29]	0.11 [0.01, 0.21]	0.12 [0.01, 0.22]	0.21 [0.09, 0.33]
FFN-pt	0.19 [–0.01, 0.36]	0.21 [0.08, 0.34]	0.11 [–0.00, 0.19]	0.23 [0.11, 0.34]
CNN-pt	0.28 [0.12, 0.41]	0.34 [0.18, 0.47]	0.26 [0.13, 0.37]	0.34 [0.21, 0.45]
GNN-pt	0.28 [0.15, 0.39]	0.31 [0.13, 0.46]	0.23 [0.10, 0.35]	0.35 [0.16, 0.51]
RF-nd	0.19 [0.08, 0.29]	0.16 [0.05, 0.26]	0.26 [0.17, 0.34]	0.23 [0.09, 0.34]
FFN-nd	0.15 [0.02, 0.26]	0.14 [–0.01, 0.26]	0.11 [–0.02, 0.24]	0.14 [–0.02, 0.27]
CNN-nd	0.13 [–0.01, 0.25]	0.19 [0.02, 0.31]	0.24 [0.10, 0.36]	0.16 [–0.01, 0.29]
**GNN-nd**	0.17 [0.03, 0.28]	0.19 [0.04, 0.33]	**0.31 [0.18, 0.43]**	0.29 [0.14, 0.41]
FFN-pt-nd	0.21 [0.07, 0.35]	0.25 [0.12, 0.37]	0.16 [0.03, 0.28]	0.26 [0.14, 0.37]
**CNN-pt-nd**	**0.29 [0.15, 0.42]**	**0.35 [0.20, 0.47]**	0.28 [0.15, 0.40]	**0.36 [0.21, 0.49]**
GNN-pt-nd	0.28 [0.14, 0.40]	0.31 [0.17, 0.43]	0.24 [0.10, 0.35]	0.35 [0.20, 0.47]

The 95% confidence intervals, presented in square brackets, were estimated from 1,000 bootstrap iterations on the test set. The best models for each task are highlighted in bold. *ni*, no-imaging; *pt*, models with pre-training; *nd*, use of neuropsychology and demographic information.

Across all cognitive domains, with the exception of visuospatial, models that incorporated the pre-training step generally outperformed those without it. Moreover, CNN-based models consistently yielded the highest predictive performance, followed by GNN-based models, while tabular models (FFN and RF) exhibited the weakest results.

**Figure 3:**
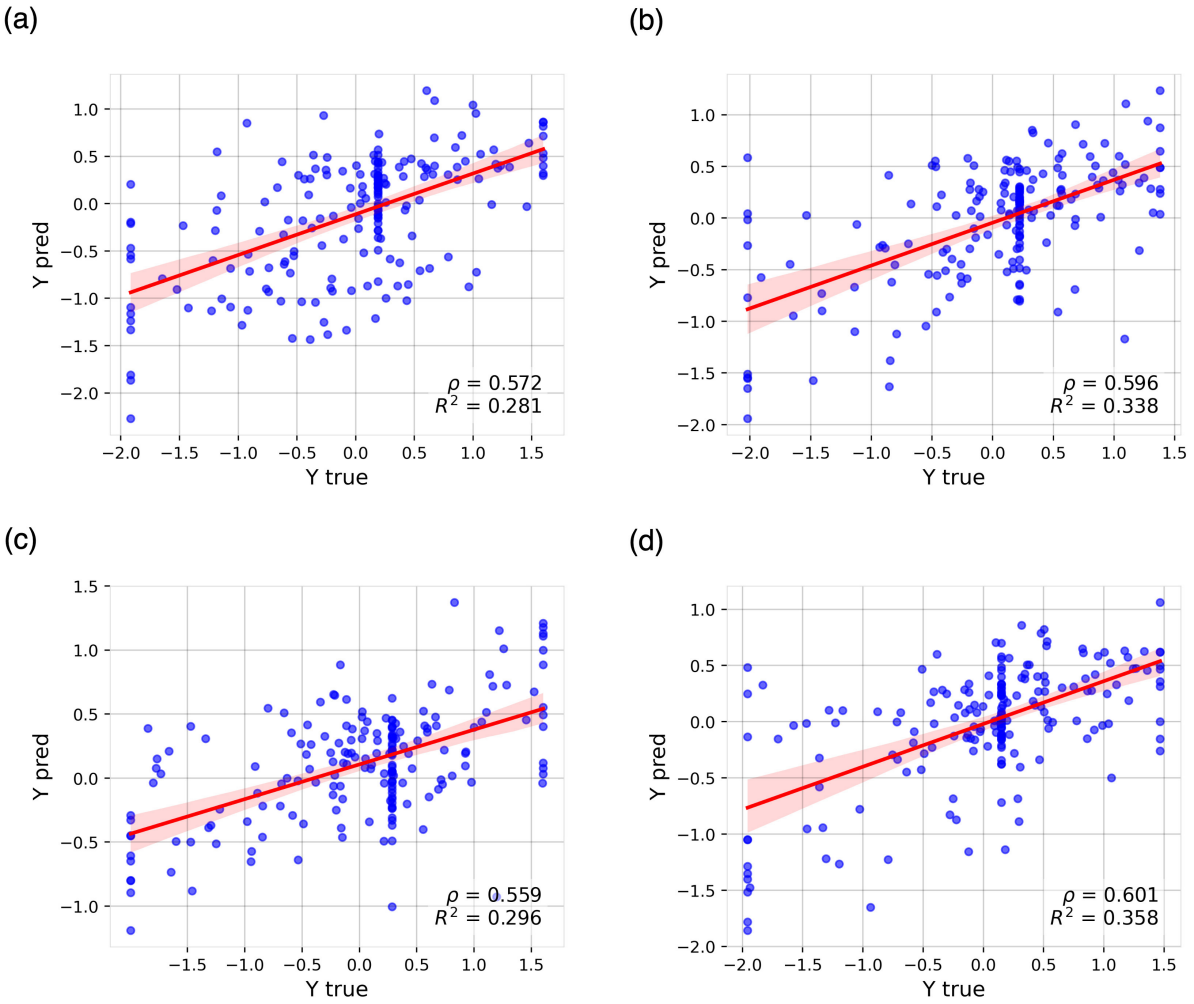
Predicted values (y-axis) vs. actual values (x-axis) from the best-performing regression models for predicting cognitive decline across the evaluated cognitive domains: **(a)** memory, **(b)** language, **(c)** visuospatial abilities, and **(d)** executive functions. Cognitive decline ratios are expressed as z-scores. The plot includes the regression line along with 95% confidence intervals. ρ, Pearson correlation coefficient; *R*^2^, coefficient of determination.

In the memory domain, the CNN-pt-nd achieved the best results, with an EV of 0.294 and a correlation of 0.572 between predicted and actual scores (Figure 3a). Other models with strong performance—including CNN-pt, GNN-pt-nd, and GNN-pt—also exceeded an EV of 0.25. These models clearly outperformed the RF baseline using only neuropsychological and demographic data (EV 0.062), with the remaining models ranging between EVs of 0.073 and 0.212.

In the language domain, the CNN-pt-nd again led in performance (EV 0.346, correlation 0.596; Figure 3b). As in the memory domain, CNN and GNN models achieved EVs above 0.3, while FFN models with pre-training showed moderate performance (EV >0.2). The rest of the models performed poorly, with EVs below 0.2.

In the visuospatial domain, models also achieved EVs above 0.3 (Figure 3c), although the advantage of pre-training was less evident. Interestingly, the RF model excluding neuroimaging data performed competitively (EV 0.259), ranking among the top models. CNN variants—CNN-pt-nd, CNN-pt, and CNN-nd—followed closely (EVs 0.277, 0.255, and 0.239, respectively). In contrast, models trained solely on neuroimaging features performed poorly (EV < 0.124).

Finally, the executive functions domain yielded the highest EV values across all tasks (Figure 3d). The CNN-pt-nd again achieved the best performance (EV 0.360, correlation 0.601), followed by the GNN-pt, which attained the highest correlation (0.611) and an EV of 0.354. Models leveraging pre-training or neuropsychological and demographic information (pt, nd, pt-nd) consistently outperformed the remaining configurations (EV >0.25). Conversely, models without pre-training or based on tabular inputs achieved the worst results. The RF-ni model, which excluded neuroimaging, showed the lowest predictive accuracy.

### 3.5 Qualitative prediction of cognitive decline

The results and comparisons of the top-performing models for qualitative classification of cognitive decline are presented in Table 3. Further performance metrics are presented in Appendix 6.

**Table 3:** Matthews correlation coefficient of classification models predicting binary measures of cognitive decline.

**Model**	**Memory**	**Language**	**Visuospatial**	**Executive**
RF-ni	0.31 [0.18, 0.44]	0.30 [0.16, 0.43]	0.32 [0.19, 0.45]	0.31 [0.18, 0.44]
RF	0.38 [0.23, 0.52]	0.38 [0.23, 0.52]	0.23 [0.10, 0.37]	0.34 [0.20, 0.45]
FFN	0.32 [0.16, 0.45]	0.39 [0.25, 0.52]	0.28 [0.15, 0.41]	0.36 [0.21, 0.51]
CNN	0.36 [0.22, 0.51]	0.35 [0.22, 0.48]	0.17 [0.02, 0.30]	0.37 [0.24, 0.49]
GNN	0.34 [0.22, 0.45]	0.39 [0.24, 0.52]	0.29 [0.15, 0.41]	0.33 [0.20, 0.46]
FFN-pt	0.38 [0.24, 0.50]	0.47 [0.33, 0.59]	0.40 [0.28, 0.54]	0.41 [0.29, 0.53]
CNN-pt	0.54 [0.42, 0.65]	0.57 [0.44, 0.67]	0.35 [0.22, 0.49]	0.46 [0.33, 0.57]
**GNN-pt**	0.52 [0.37, 0.64]	0.51 [0.37, 0.63]	0.32 [0.18, 0.44]	**0.49 [0.34, 0.62]**
RF-nd	0.39 [0.25, 0.52]	0.41 [0.27, 0.55]	0.23 [0.11, 0.36]	0.39 [0.25, 0.52]
FFN-nd	0.35 [0.19, 0.49]	0.40 [0.26, 0.53]	0.21 [0.08, 0.34]	0.41 [0.27, 0.53]
CNN-nd	0.31 [0.17, 0.44]	0.36 [0.22, 0.49]	0.29 [0.17, 0.42]	0.33 [0.20, 0.46]
**GNN-nd**	0.36 [0.21, 0.50]	0.37 [0.23, 0.51]	**0.42 [0.28, 0.55]**	0.36 [0.21, 0.48]
FFN-pt-nd	0.43 [0.28, 0.57]	0.50 [0.35, 0.63]	0.32 [0.19, 0.45]	0.44 [0.29, 0.56]
**CNN-pt-nd**	0.48 [0.36, 0.61]	**0.61 [0.48, 0.72]**	0.37 [0.24, 0.50]	0.45 [0.33, 0.57]
**GNN-pt-nd**	**0.56 [0.43, 0.68]**	0.56 [0.43, 0.68]	0.38 [0.24, 0.51]	0.48 [0.34, 0.60]

The 95% confidence intervals, presented in square brackets, were estimated from 1,000 bootstrap iterations on the test set. The best models for each task are highlighted in bold. *ni*, no-imaging; *pt*, models with pre-training; *nd*, use of neuropsychology and demographic information.

Across the cognitive domains of memory, language, and executive functions, models that incorporated the pre-training strategy consistently outperformed those that did not. In particular, GNNs and CNNs demonstrated superior predictive performance compared to models based solely on tabular data.

**Figure 4:**
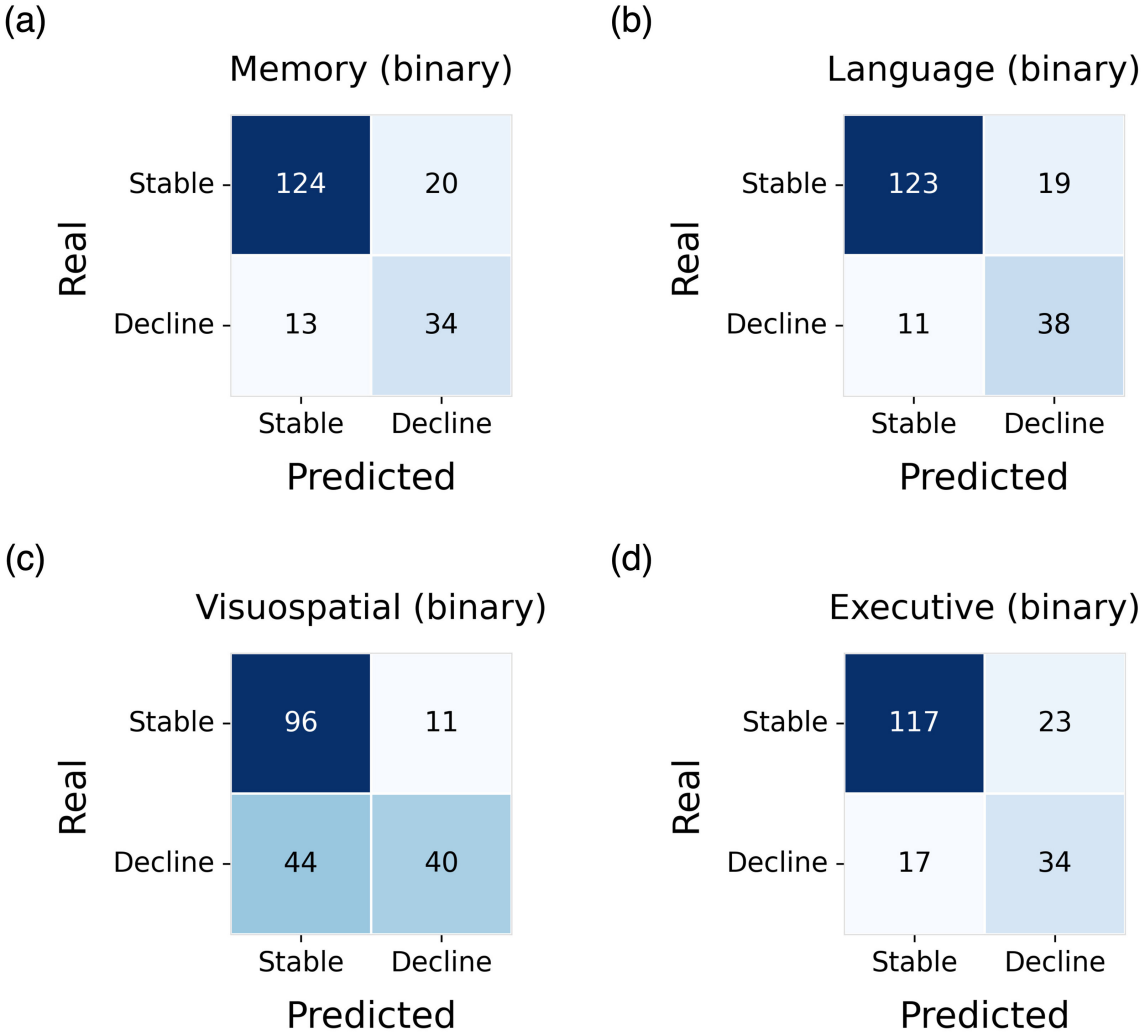
Confusion matrices obtained on the test set for the identification of decliners and stable subjects. Results are shown for the best-performing model in each cognitive domain: **(a)** memory, **(b)** language, **(c)** visuospatial abilities, and **(d)** executive functions.

The highest predictive performance was observed in the language domain, where the pre-trained GNN (GNN-pt) achieved an AUC of 0.882, with a sensitivity and specificity of 86.62% and 77.55% respectively (Figure 4b). For the memory domain, the best-performing model was the GNN-pt-nd, which reached an AUC of 0.840, with a sensitivity of 86.11% and a specificity of 72.34% (Figure 4a). In the executive functions domain, the highest performance was obtained by the GNN-pt model, achieving an AUC of 0.814, with sensitivity and specificity values of 83.57% and 66.67% (Figure 4d).

In contrast, the visuospatial abilities domain yielded the lowest predictive performance. Here, the best result was obtained using the GNN without pre-training (GNN-nd), with an AUC of 0.691, sensitivity of 89.72%, and specificity of 47.62% (Figure 4c).

### 3.6 Architectural analysis of the best models

This section analyzes the architectures derived from hyperparameter optimization for the models that demonstrated superior performance, specifically those based on GNNs and CNNs with pre-training.

For the CNN-based models, the highest-performing configurations consistently followed the architecture proposed in Garćıa-Gutiérrez et al. (2024b). This architecture comprises eight convolutional layers, incorporating normalization, max pooling, and dropout. Notably, instance normalization significantly outperformed batch normalization across experiments. The top-performing CNN models for both the MRI and FDG imaging modalities adopted this architecture. Each of these CNN models contained approximately 150K trainable parameters.

In contrast, the best-performing pre-trained CNN model for the AV45 modality was based on the DenseNet architecture. This configuration included three bottleneck blocks with 4, 6, and 8 bottleneck layers, respectively, interleaved with transition layers. A growth rate of 4 and a compression factor of 1.0 were used, alongside instance normalization. The total number of trainable parameters in this model was approximately 215K.

For the GNN-based models, hyperparameter optimization favored shallow architectures, typically comprising six message-passing layers and a high channel dimensionality (128 channels). Moreover, the use of jumping knowledge connections with concatenation, combined with average pooling over node embeddings, demonstrated superior performance. Among the evaluated models, the GAT architecture yielded the best results for the MRI and FDG modalities, whereas GIN proved most effective for the AV45 modality.

Regarding λ, no consistent trend was observed. For MRI, the best-performing configuration employed a λ value of 0.4 (sparsest graph), while the optimal values for FDG and AV45 were 0.3 and 0.2, respectively (densest graph). The approximate number of trainable parameters was 315K for GAT-based models and 250K for GIN-based models.

### 3.7 Pre-training evaluation

The results presented in Sections 3.1–3.5 indicate that the proposed pre-training strategy consistently yielded substantial performance improvements across all evaluated tasks.

To further examine the influence of pre-training dataset size on model performance—and, by extension, the scalability of the strategy with increased data availability—learning curves of the CNN-pt-nd model were analyzed.

**Figure 5:**
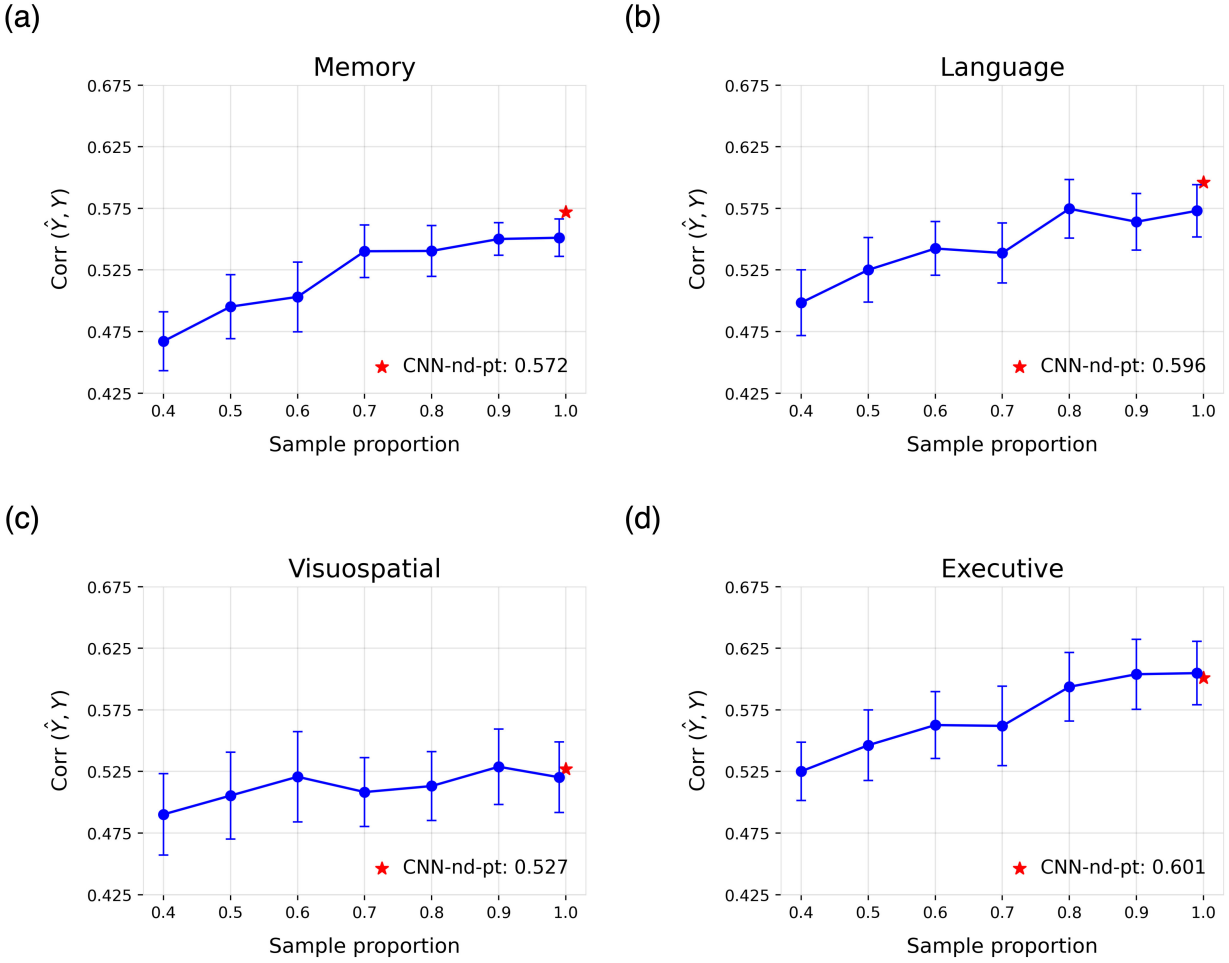
Learning curves for the cognitive domains of **(a)** memory, **(b)** language, **(c)** visuospatial abilities, and **(d)** executive functions. Each curve shows the mean correlation and standard deviation across 100 runs of the CNN-pt-nd model on the test set, obtained using models pre-trained with varying proportions of the original pre-training dataset. The reported result of the best model trained on all data is highlighted in red.

Specifically, 100 models were pre-trained for each modality using varying proportions of the original pre-training dataset, followed by fine-tuning as described in Section 2.6. In this analysis, no hyperparameter optimization was performed; instead, the configuration that demonstrated the most consistent performance across tasks was selected. Moreover, learning curves were generated exclusively for the quantitative prediction of cognitive decline across different cognitive domains. The resulting curves, assessed in terms of mean correlation, are presented in Figure 5.

## 4 Discussion

This study addresses the pressing need for domain-specific prediction of cognitive decline in AD, moving beyond conventional global cognitive assessments. Despite the inherent challenges associated with neuroimaging-based AI applications, such as high data dimensionality and limited sample sizes, our framework demonstrates a strong capacity for modeling individualized cognitive trajectories across distinct cognitive domains. By incorporating a dedicated pre-training step, we significantly enhanced predictive performance, surpassing conventional baselines in capturing the heterogeneous patterns of decline in memory, language, executive function, and visuospatial abilities.

We first evaluated the classification capabilities of our models to assess the reliability of the proposed framework. To this end, we compared our approach with benchmark neuroimaging models reported in recent studies on clinical diagnosis prediction (Sharma et al., 2023; Kaur and Sachdeva, 2025). The resulting weighted macro–F1 scores, 0.745 for the 2-year and 0.779 for the 4-year forecasts, place our models within the range of high-performing neuroimaging classifiers. Most misclassifications occurred between CN and MCI groups (Figures 2c–d), which reflects the well-documented clinical ambiguity of these transitional stages (Petersen, 2004).

Furthermore, although the framework was not specifically optimized for conversion prediction, it achieved robust performance in identifying converters within four years (AUC = 0.909), consistent with current state-of-the-art results (Ansart et al., 2021; Sharma et al., 2023; Lee et al., 2024; Yang et al., 2025). Performance at two years (AUC = 0.772) was comparatively lower, likely reflecting diagnostic uncertainty and the limited number of conversion events in brief follow-up intervals (Farias et al., 2009; Garćıa-Gutiérrez et al., 2024b). These findings underscore the robustness of our multimodal modeling approach and align with previously reported AUCs ranging from 0.78 to 0.96 in longitudinal AD studies (Abrol et al., 2020; Wang et al., 2024; Dao et al., 2025).

In these diagnostic classification problems, the superior performance observed at the four-year horizon likely stems from intrinsic features of AD progression. Longer follow-up periods mitigate diagnostic noise and class imbalance, allowing disease-related patterns to manifest more clearly, whereas short-term variability is often influenced by individual factors such as cognitive reserve and comorbidities (Farias et al., 2009; Duara and Barker, 2022; Abdelnour et al., 2022). Consequently, extended observation windows provide more stable, pathology-driven predictions.

While these diagnostic results support the validity of our framework, the primary contribution of this study lies in advancing domain-specific modeling of cognitive decline rather than establishing new diagnostic benchmarks. Recent evidence suggests that conversion-prediction models may have reached a performance plateau, indicating that future progress toward clinical translation will depend more on expanding sample sizes, improving cross-cohort generalization, and enhancing model reliability (El-Sappagh et al., 2023). Within this context, domain-specific modeling of cognitive decline remains comparatively underexplored, despite its critical relevance for early intervention and clinical decision-making (Abdelnour et al., 2022; Hampel et al., 2022; Devanarayan et al., 2025).

Accordingly, our findings demonstrate that patterns of cognitive decline can be inferred from neuroimaging data, particularly through spatial features extracted from brain images. Although the models explained a moderate proportion of the variance in cognitive decline (29.4%–36.0%), the predicted trajectories were well correlated with the observed outcomes (correlation > 0.55). In the binary classification setting, the models also exhibited strong performance in identifying individuals whose decline deviated from normative aging trajectories. Notably, AUC values exceeded 0.83 in the memory, language, and executive function domains.

Visuospatial abilities constituted the main exception to these trends. In this domain, performance dropped markedly when cognitive decline was binarized, despite regression models achieving errors comparable to the other domains. This discrepancy is likely driven by the high sensitivity of the binary label to the normative cut-off, because annual change rates cluster near the threshold and small score fluctuations can flip individuals between *stable* and *decliner* categories, adding label noise. In addition, the visuospatial composite relies on tests with limited score range and pronounced ceiling and floor effects, particularly among CN and MCI populations (Kueper et al., 2018). This further increases the risk that subtle variations are misclassified as categorical change rather than genuine trajectory shifts. Future work should incorporate more fine-grained visuospatial measures and larger normative samples to reduce threshold sensitivity and stabilize qualitative predictions.

To date, few studies based on multimodal neuroimaging have modeled cognitive decline. Moreover, most existing works rely on tabular data and are typically limited to neuropsychological assessments of global cognition, such as the MMSE (Zhang et al., 2012; Huang et al., 2016; Franzmeier et al., 2020; Lei et al., 2020; Dansson et al., 2021; Lei et al., 2022; Maheux et al., 2023; Devanarayan et al., 2024). For example, in Franzmeier et al. (2020), the authors used MRI, FDG, and amyloid-PET data to predict cognitive decline rates—similar to those defined in this study—explaining approximately 25% of the variance in global cognition and memory. Similarly, in studies such as Dansson et al. (2021) and Devanarayan et al. (2024), cognitive decline was modeled in terms of MMSE score changes at two and four years, achieving R^2^ values of 0.325 and 0.228, respectively. In Zhang et al. (2012), correlation coefficients of 0.697 and 0.739 were reported for MMSE and ADAS-Cog. Notably, one of the studies most closely related to our work modeled cognitive decline across the domains of memory, language, executive function, and visuospatial abilities, yielding correlation values between 0.42 and 0.50, lower than those obtained in the present study (Moradi et al., 2025).

In addition, several studies have focused on modeling future cognitive scores—i.e., point estimates of the expected value at a single time point—for tests such as the ADAS-Cog, CDR, or MMSE, usually reporting R^2^ values above 0.65 (Huang et al., 2016; Lei et al., 2020, 2022; Maheux et al., 2023). However, this point-estimate approach presents limitations when assessing the true predictive capacity of the models, as baseline scores already account for a substantial proportion of the variance. As a result, the reported explained variance may be misleading. For instance, in groups where cognitive scores remain relatively stable over time, a naive strategy that simply reproduces baseline scores can yield high correlations with future outcomes. This highlights one of the key strengths of the approach presented here, which focuses instead on modeling general trends in cognitive trajectories rather than static future scores.

Our analysis also yielded several technical insights. First, models based on CNNs and GNNs consistently outperformed alternative approaches. Incorporating spatial features was particularly advantageous for complex tasks such as cognitive decline prediction and surpassed the performance of the tabular AI models that we implemented. These gains validate the extra implementation effort required to ingest volumetric or surface-based inputs and suggest that future neuro-AI pipelines should treat spatial encoding as a core design criterion rather than an optional add-on.

Second, although CNNs achieved the highest overall performance, their advantage over GNNs was often marginal, indicating that the increased memory demands of CNNs may not always be justified. In scenarios where hospitals deploy models on embedded hardware, or where cloud inference is billed by runtime and memory usage, the difference in resource usage between GNNs and CNNs may outweigh the slight accuracy advantage of CNNs, making GNNs the more pragmatic choice.

Finally, the proposed pre-training strategy consistently enhanced performance across tasks. Moreover, the learning curves suggest that larger pre-training datasets could lead to further improvements, which offer promising opportunities for model development in settings with limited data.

Within this context, the present study represents a substantial advancement over previous work by modeling cognitive decline across specific domains rather than relying solely on global cognition scores. This design enables a more granular characterization of neurodegenerative trajectories, an aspect of considerable clinical relevance.

Methodologically, our work extends previous multimodal fusion approaches (Zhang et al., 2023; Yu et al., 2024; Abdelaziz et al., 2025) by (i) shifting the focus from purely diagnostic endpoints to domain-specific cognitive trajectories, (ii) integrating several imaging modalities together with rich neuropsychological profiles within a unified comparative framework across FFN, CNN, and GNN backbones, and (iii) introducing a modality-specific multi-task pre-training stage that produces trajectory-aware embeddings for subsequent late fusion.

From an applied standpoint, accurately predicting cognitive decline in AD yields several clinically significant benefits. First, it enables genuinely personalized, dynamic therapy, since clinicians could adjust pharmacological or behavioral interventions in real time as the model detects accelerating progression (Jutten et al., 2021). Second, reliable forecasts support advance-care planning, giving patients and families evidence-based timelines for financial, legal, and caregiving decisions. Third, at the operational level, trajectory prediction helps healthcare systems triage high-risk individuals for closer surveillance and allocate scarce resources, such as specialist memory-clinic appointments, more efficiently (Alzheimer's Association, 2024).

In the context of drug development, prognostic models could facilitate tighter enrichment of clinical-trial cohorts and more informed endpoint selection. For example, endpoints could be tailored to the specific cognitive domains most likely to decline within a given cohort, thereby lowering required sample sizes and increasing power to detect disease-modifying effects (Jutten et al., 2021; Duara and Barker, 2022). Ultimately, domain-level prediction of cognitive decline may be particularly valuable for informing personalized cognitive therapies that target the most at-risk cognitive functions. Collectively, these capabilities have the potential to advance both everyday clinical care and translational AD research.

Despite these promising results, the study has several limitations. Most notably, the predictive performance for cognitive decline remains moderate, which reflects the complexity of neurodegenerative processes. These processes are shaped by multifactorial and stochastic interactions among genetic, environmental, and lifestyle factors, resulting in substantial interindividual variability that is challenging to model with limited sample sizes (Alzheimer's Association, 2024). To improve model accuracy, increasing cohort sizes and incorporating complementary data sources, such as genetic profiles or plasma biomarkers, will be essential. In this context, federated learning offers a scalable and privacy-preserving approach to integrate multi-site data and enhance model generalizability (Rieke et al., 2020).

From a methodological perspective, our comparative non-deep learning baseline was an RF model, and we did not include other non-deep multimodal baselines or hybrid ensembles. Given that the available literature on this task is limited and predominantly deep-learning oriented (Kaur and Sachdeva, 2025), a more comprehensive benchmarking analysis should be undertaken in future work to better contextualize the state of the art in domain-level cognitive-decline prediction.

Another widely recognized limitation that applies to this study, and to much of the current literature, is the difficulty of generalizing models across cohorts. Our analyses were restricted to the ADNI dataset, limiting the findings to this specific population (Samper-González et al., 2017). This constraint may reduce the clinical applicability of the results, as research cohorts such as ADNI often differ from real-world clinical populations in demographic composition, disease spectrum, and imaging protocols. This issue is particularly relevant given the known variability in the demographics and clinical presentation of AD. Therefore, external validation on independent, multi-site cohorts (e.g., AIBL, OASIS) and, ideally, prospective clinical studies conducted in real-world settings will be essential to establish robustness, generalizability, and clinical utility. Future work should prioritize evaluating the proposed framework in such diverse cohorts as a necessary step toward translational deployment.

Future studies should also investigate whether incorporating longitudinal information, such as serial neuroimaging scans or repeated cognitive assessments, provides meaningful improvements in predictive performance. However, acquiring such data is logistically challenging, because repeated MRI or PET sessions are costly, time-consuming, and consequently rare in routine clinical practice. Rigorous analyses will therefore be required to determine whether the incremental predictive gains offered by longitudinal data justify the substantial logistical and financial burden associated with their acquisition.

Finally, future research should assess the biological validity of model predictions using explainable AI (XAI) techniques tailored to neuroimaging. Demonstrating that *post hoc* attribution methods (e.g., saliency maps, attention weights, or counterfactual lesioning) assign importance to neuroanatomically plausible regions would substantially strengthen the claim that the model leverages disease-relevant signals (Arrieta et al., 2020; Bloch et al., 2024). While such interpretability analyses were beyond the scope of the present study, which primarily focused on establishing predictive performance, they constitute a critical avenue for future work. Supporting the feasibility of this approach, previous work from our group has shown that XAI pipelines in related neuroimaging contexts can produce stable attributions in canonical disease-relevant circuits (Garćıa-Gutiérrez et al., 2024b). Conducting these analyses is essential, as they can help clinicians understand the rationale behind model predictions and thereby foster clinical trust, improve patient safety, and facilitate eventual regulatory evaluation.

## 5 Conclusion

In conclusion, we developed AI-based models using multimodal neuroimaging data and systematically compared alternative information-representation strategies to predict domain-specific cognitive decline in Alzheimer's disease. Theoretically, our findings show that spatially encoded multimodal features can capture individualized trajectories across memory, language, executive, and visuospatial domains, moving beyond conventional global cognition scores and point-estimate forecasting. Practically, our approach provides a scalable tool for stratifying patients according to their risk of accelerated decline in specific cognitive domains, thereby supporting personalized intervention planning, optimized resource allocation, and the enrichment and endpoint selection of clinical trials. By maximizing the use of all available neuroimaging information to generate domain-level trajectory predictions, this study offers both a conceptual and methodological advance in multimodal modeling and contributes to the development of clinically meaningful prognostic tools for neurodegenerative diseases.

## Data Availability

The datasets generated and/or analyzed during the current study are available from the ADNI database (https://adni.loni.usc.edu/). The code used for this research is available at: https://github.com/FernandoGaGu/Domain-cognitive-decline-prediction. Further inquiries can be directed to the corresponding author.
