# Supplementary material for: Deep multimodal learning for domain-level cognitive decline prediction in Alzheimer's disease
Source: Front Artif Intell. 2025 Dec 17;8:1731062. doi: 10.3389/frai.2025.1731062 (PMC12753913; doi:10.3389/frai.2025.1731062)
Supplement: Supplementary file 1 [file Data_Sheet_1.pdf]

# Supplementary material. Deep Multimodal Learning for Domain-level Cognitive Decline Prediction in Alzheimer's Disease

## Appendix 1. Analysis of processed neuroimaging information and anomaly detection

Outlier and anomaly detection techniques were applied to the preprocessed neuroimaging data to identify and remove scans potentially affected by acquisition protocol deviations or preprocessing pipeline errors.

Detection was performed at the level of region of interest (ROI) using features derived from the Automated Anatomical Labeling (AAL) atlas parcellation [1]. For magnetic resonance imaging (MRI) data, gray matter volumes were extracted, while for [$^{18}$F]-fluorodeoxyglucose (FDG) and [$^{18}$F]-florbetapir (AV45) positron emission tomography (PET) images, the median intensity values within each ROI were used. For PET images, the standardized uptake value ratios (SUVRs) normalized according to the value of the cerebellum were considered [2].

First, statistical filtering was employed to remove outlier images. An image was classified as an outlier if more than 20% of its ROIs fell outside 2.5 times the interquartile range of the entire dataset.

Subsequently, the Isolation Forest algorithm was applied using the parameters specified in Table A1 [3]. The Scikit-learn implementation of the algorithm was used (v1.6.1) [4]. Based on the contamination scores produced by the algorithm, images were flagged as anomalous if their score exceeded the mean by more than three standard deviations. Using this procedure, 5.41%, 6.02%, and 2.46% of the MRI, FDG, and AV45 images, respectively, were identified as containing artifacts.

Table A1: Selected hyperparameters for the Isolation Forest algorithm.

| Parameter | Value | Description |
|---|---|---|
| n_estimators | 600 | Number of adjusted trees. |
| max_samples | 0.75 | Proportion of samples used to fit each tree. |
| max_features | 0.75 | Proportion of features used to fit each tree. |
| bootstrap | False | Controls whether the sampling used to construct each tree is done with replacement. |

All other unspecified parameters were left at their default values.

# Appendix 2. Imputation of missing values in the Boston Naming Test

Given the high proportion of missing values in the Boston Naming Test (BNT; greater than 20%), a different imputation strategy was employed compared to the rest of the neuropsychological assessments.

This high level of missingness in the BNT is explained by the fact that the test was no longer administered starting with the ADNI3 cohort. This pattern of missing data is therefore driven by the study design and corresponds to a missing not at random (MNAR) mechanism. In this study, however, we assume that the exclusion of the BNT was not associated with any latent characteristics of the participants. Under this assumption, it is reasonable to treat the missingness as missing at random (MAR), which allowed us to condition the values of the BNT to the rest of the neuropsychological evaluations [5].

Accordingly, BNT imputation was performed using a set of predictors, including (i) previously imputed values from other neuropsychological tests, (ii) demographic variables (age, sex, and years of education), and (iii) the current clinical diagnosis encoded as a one-hot vector. These features were used as input for a Random Forest model that was trained to predict BNT scores.

To evaluate the robustness of the imputation, we assessed the Random Forest model using five-fold cross-validation repeated ten times, using the available (non-missing) BNT scores. This procedure aimed to estimate the model's ability to infer BNT values from the selected predictors.

Experimental results from the cross-validation showed that the model achieved a correlation of 0.765 and a mean absolute error of 2.197, indicating strong predictive performance (Figure A1).

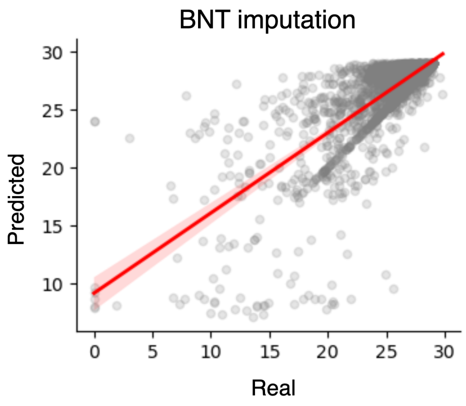


Figure A1: Actual vs. imputed Boston Naming Test (BNT) values for those individuals with known BNT values.

# Appendix 3. Computation and analysis of cognitive domain composites

Cognitive composites were computed to represent the domains of memory, language, visuospatial abilities, and executive functions. These composites were derived from neuropsychological test scores using structural equation modeling (SEM), with models specifically designed to characterize each cognitive domain. This approach captures the shared variance among multiple tests intended to assess the same latent construct, thereby providing a more reliable and domain-specific cognitive measure [6].

Following the computation of the cognitive composites, we observed that each neuropsychological test showed a high correlation with its corresponding composite, and low correlations with the others. The correlation values between the individual neuropsychological tests and the computed composites are presented in Figure A4. Additionally, the proportion of variance explained by each composite in its associated tests was high, supporting the interpretation that each composite effectively captures the shared variance among tests designed to assess the same cognitive construct (Table A2).

Finally, we observed strong correlations between the various rates of cognitive decline and diagnostic changes, providing further evidence for the consistency and validity of the cognitive decline quantification (Table A3).

Table A2: Explained variance of each of the neuropsychological tests in each associated composite.

| Domain | Test | EV domain$^a$ | EV no domain$^b$ |
|---|---|---|---|
| Executive functions | Attn num cancell | 0.62 | 0.27 |
| | TMT-a | 0.83 | 0.31 |
| | TMT-b | 0.73 | 0.34 |
| Language | BNT (tot) | 0.65 | 0.23 |
| | Category fluency | 0.74 | 0.38 |
| | Naming objects | 0.51 | 0.18 |
| | Word finding diff | 0.33 | 0.18 |
| Memory | AVLT (delayed) | 0.79 | 0.26 |
| | AVLT (recognition) | 0.57 | 0.25 |
| | AVLT (trial 1) | 0.45 | 0.23 |
| | AVLT (trial 2) | 0.70 | 0.31 |
| | AVLT (trial 6) | 0.84 | 0.29 |
| | Word recall | 0.74 | 0.44 |
| | Word recall (delayed) | 0.80 | 0.36 |
| | Word recognition | 0.51 | 0.28 |
| Visuospatial abilities | Clock copy | 0.59 | 0.24 |
| | Clock draw | 0.81 | 0.32 |
| | Constructional praxis | 0.38 | 0.18 |
| | Ideational praxis | 0.25 | 0.16 |

Abbreviations: TMT, Trail Making Test; BNT, Boston Naming Test; AVLT, Rey Auditory Verbal Learning Test.
[a] Explained variance in the target cognitive domain.
[b] Maximum explained variance by a cognitive domain other than the target domain.

Furthermore, we observed a strong alignment between the composite scores and clinical diagnostic groups (Figure A3). In all domains, individuals diagnosed with dementia showed the lowest composite scores, cognitively normal participants showed the highest scores, and individuals with mild cognitive impairment exhibited intermediate values.

Table A3: Cognitive decline statistics by domain, including quantitative and qualitative measures, and association with clinical progression.

| **Diagnosis transition** | **Quantitative** [a] | | | | **Qualitative** [b] | | | | **Decline in ≥ 2 domains** [c] |
|---|---|---|---|---|---|---|---|---|---|
| | **Memory** | **Language** | **Visuospatial** | **Executive** | **Memory** | **Language** | **Visuospatial** | **Executive** | |
| sCN (201) | 0.3 (3.9) | 0.8 (6.5) | -0.6 (12.1) | 0.4 (6.5) | 20 (10.0) | 22 (10.9) | 59 (29.4) | 30 (14.9) | 28 (13.9) |
| pCN (66) | -2.8 (5.9) | -4.2 (7.8) | -5.3 (12.5) | -1.3 (7.4) | 26 (39.4) | 23 (34.8) | 30 (45.5) | 20 (30.3) | 29 (43.9) |
| sMCI (232) | -0.1 (5.9) | -1.1 (9.7) | -2.7 (12.4) | 0.6 (8.8) | 45 (19.4) | 60 (25.9) | 95 (40.9) | 57 (24.6) | 75 (32.3) |
| pMCI (121) | -5.8 (6.5) | -9.9 (14.9) | -12.6 (18.7) | -10.1 (13.3) | 75 (62.0) | 68 (56.2) | 79 (65.3) | 72 (59.5) | 90 (74.4) |
| rMCI (33) | 3.4 (4.5) | 1.6 (6.4) | -0.8 (10.2) | 4.2 (6.0) | 1 (3.0) | 3 (9.1) | 11 (33.3) | 1 (3.0) | 2 (6.1) |

Abbreviations: sCN, stable cognitively normal; pCN, progressive cognitively normal; sMCI, stable mild cognitive impairment; pMCI, progressive mild cognitive impairment; rMCI, mild cognitive impairment reverting to cognitively normal.

[a] Mean value and standard deviation of the quantitative version of cognitive decline. Expressed in cognitive domain units per year.

[b] Number and percentage of subjects identified as decliners.

[c] Number and percentage of subjects showing decline in two or more cognitive domains.

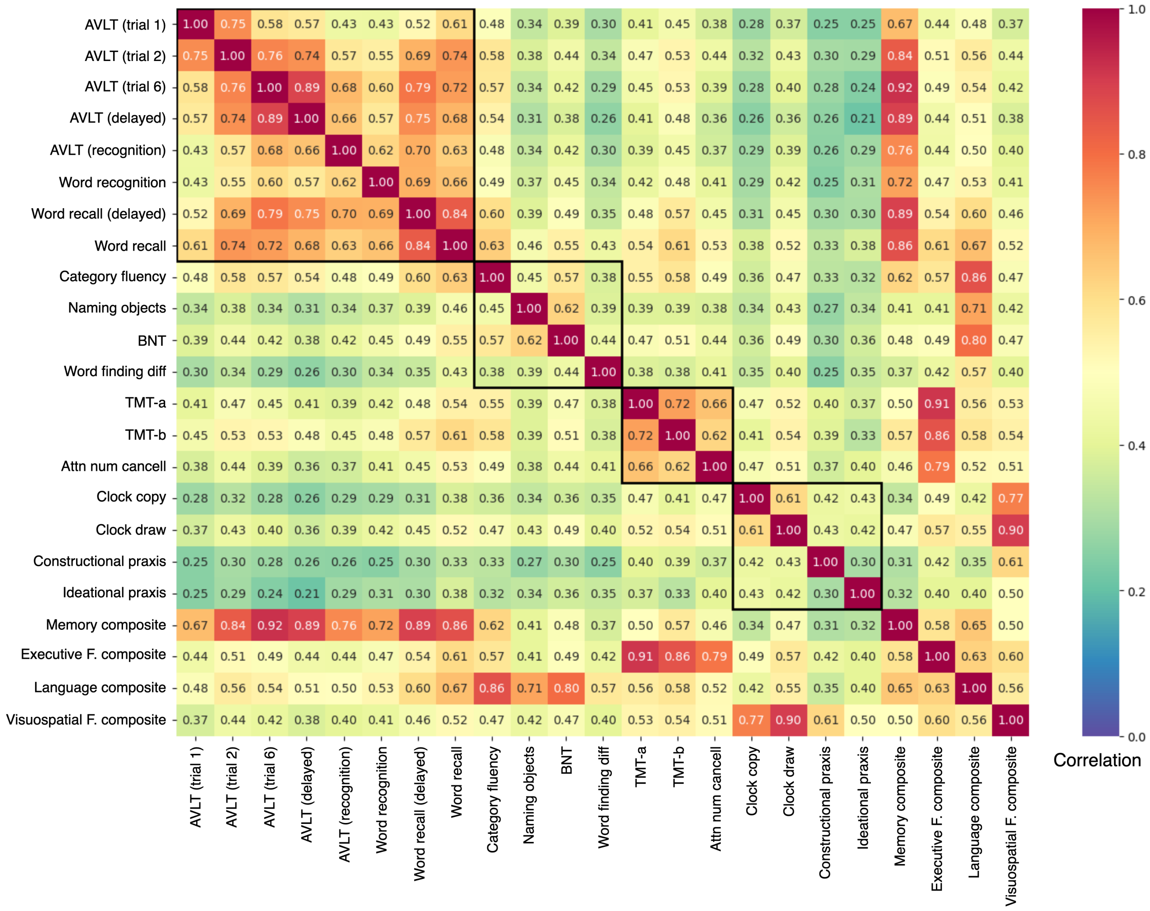


Figure A2: The Pearson correlation values show the relationships between the different neuropsychological tests and the calculated composites. In the correlation graph, the squares group the tests belonging to the same cognitive domain.

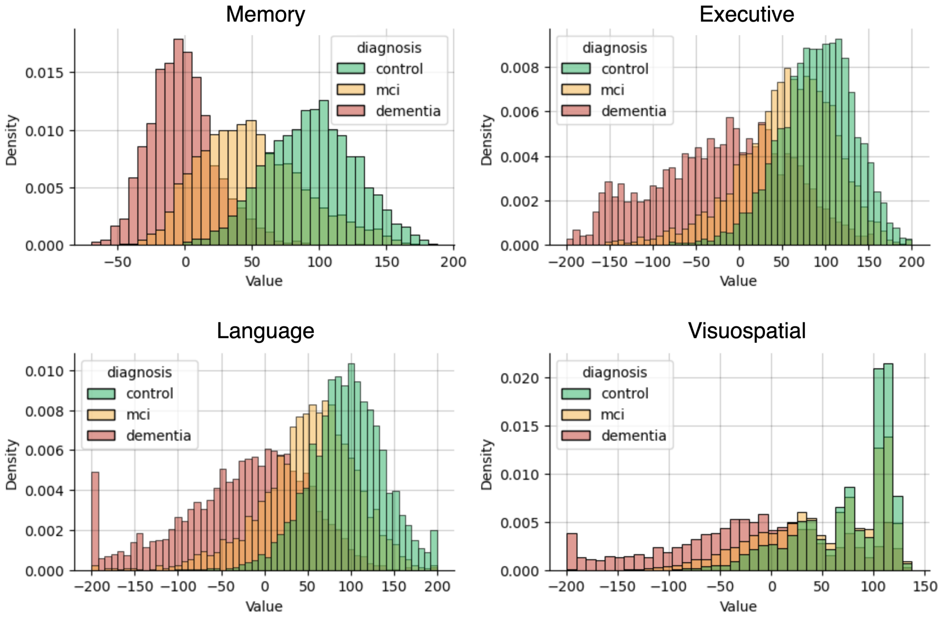


Figure A3: Distribution of the values of the different neuropsychological composites calculated according to clinical diagnosis. Abbreviations: mci, mild cognitive impairment.

# Appendix 4. Hyperparameter optimization of the models

In this study, various hyperparameter combinations were explored for different model architectures, including feed-forward networks (FFNs), convolutional neural networks (CNNs), and graph neural networks (GNNs). Hyperparameter optimization was conducted using a grid search approach, and the specific configurations evaluated are detailed in Tables A4–A6. Based on the results obtained from this optimization, the final architectures of the models presented in the main manuscript were designed.

Table A4: Hyperparameters considered for feed-forward networks models.

| Parameter | Values |
| --- | --- |
| Layer architecture[a] | [100, 75, 50]<br>[200, 100, 50]<br>[300, 200, 100, 50]<br>[400, 200, 75]<br>[400, 300, 200, 75]<br>[400, 300, 200, 100, 50]<br>[400, 300, 200, 100, 50] |
| Activation function | {ReLU, LeakyReLU, SiLU} |
| Dropout rate | {0.0, 0.1, 0.15} |
| Batch normalization | {True, False} |

[a] The lists show the number of neurons per layer for each of the tested architectures.

Table A5: Hyperparameters considered for convolutional neural networks models.

| Model scaffold[a] | Parameter | Values |
| --- | --- | --- |
| DenseNet | Model architecture[b] | L=4,6,8; k=4; $\theta$=0.5<br>L=4,6,8; k=8; $\theta$=0.5<br>L=4,6,8; k=4; $\theta$=1.0<br>L=4,6,8; k=8; $\theta$=1.0<br>L=8,12,16; k=4; $\theta$=0.5<br>L=8,12,16; k=8; $\theta$=0.5<br>L=8,12,16; k=4; $\theta$=1.0<br>L=8,12,16; k=8; $\theta$=1.0 |
| DenseNet | Init channels | {16, 32} |
| Simple | Number of layers[c] | {6, 8, 10} |
| Simple/DenseNet | Normalization layer | {BatchNorm3d, InstanceNorm3d} |
| Simple/DenseNet | Dropout rate | {0.0, 0.15} |

[a] Indicates to which type of model each parameter corresponds.
[b] L, indicates the number of bottleneck blocks and layers; k, growth rate; $\theta$, compression factor.
[c] Besides the number of convolutional blocks tested, the number of channels and size of the filters were manually adjusted for each configuration.

Table A6: Hyperparameters considered for graph neural networks models.

| Parameter | Values |
|---|---|
| Jumping knowledge | {last, cat} |
| Graph pooling | {mean, sum} |
| Message-passing layers | {4, 8, 12} |
| Hidden channels | {32, 64, 128, 256} |
| $\lambda$ (Graphical lasso) | {0.1, 0.2, 0.3, 0.4} |

# Appendix 5. Training and validation loss curves of the best models during fine-tuning

Figure A4 shows the training and validation loss curves of the best models during fine-tuning. These curves show how the loss values change across epochs for the training and validation sets. The best model checkpoint for each configuration was selected based on minimum validation loss.

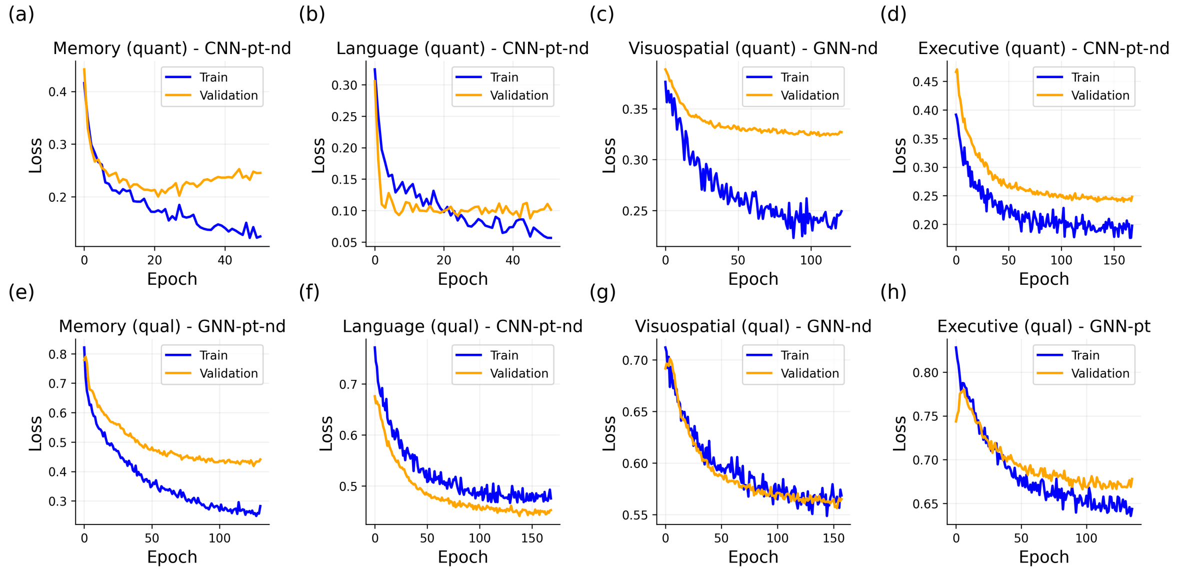


Figure A4: Learning curves of the best-performing models developed to predict cognitive decline both quantitatively (label *quant*) and qualitatively (decliner vs. stable, label *qual*). Abbreviations: CNN, convolutional neural network; GNN, graph neural network; *pt*, models with pre-training; *nd*, use of neuropsychological and demographic information.

# Appendix 6. Model performance analysis

This appendix contains the results of the best models for each of the problems not included in the main manuscript. These include diagnostic problems at two and four years, conversion to dementia at two and four years, and prediction of diagnostic transitions. Additionally, correlation coefficients for continuous cognitive decline prediction and area under the curve (AUC) values for binary classification are reported.

Table A7: Multiclass diagnostic prediction at two years.

| Model | MCC | F1-score |
|---|---|---|
| RF-ni | 0.443 | 0.669 |
| RF | 0.101 | 0.460 |
| FFN | 0.126 | 0.481 |
| CNN | 0.178 | 0.504 |
| GNN | 0.222 | 0.544 |
| FFN-pt | 0.376 | 0.628 |
| CNN-pt | 0.507 | 0.707 |
| GNN-pt | 0.483 | 0.695 |
| RF-nd | 0.478 | 0.677 |
| FFN-nd | 0.331 | 0.604 |
| CNN-nd | 0.439 | 0.665 |
| GNN-nd | 0.433 | 0.667 |
| FFN-pt-nd | 0.425 | 0.661 |
| CNN-pt-nd | 0.580 | 0.745 |
| GNN-pt-nd | 0.557 | 0.736 |

Abbreviations: MCC, Matthews correlation coefficient; RF, random forest; FFN, feed-forward network; CNN, convolutional neural network; GNN, graph neural network; *ni*, no-imaging; *pt*, models with pre-training; *nd*, use of neuropsychology and demographic information.

Table A8: Multiclass diagnostic prediction at four years.

| Model | MCC | F1-score |
|---|---|---|
| RF-ni | 0.429 | 0.653 |
| RF | 0.323 | 0.561 |
| FFN | 0.294 | 0.556 |
| CNN | 0.240 | 0.521 |
| GNN | 0.334 | 0.597 |
| FFN-pt | 0.490 | 0.688 |
| CNN-pt | 0.622 | 0.764 |
| GNN-pt | 0.529 | 0.717 |
| RF-nd | 0.396 | 0.627 |
| FFN-nd | 0.409 | 0.632 |
| CNN-nd | 0.468 | 0.672 |
| GNN-nd | 0.444 | 0.663 |
| FFN-pt-nd | 0.565 | 0.729 |
| CNN-pt-nd | 0.646 | 0.779 |
| GNN-pt-nd | 0.568 | 0.736 |

Abbreviations: MCC, Matthews correlation coefficient; RF, random forest; FFN, feed-forward network; CNN, convolutional neural network; GNN, graph neural network; *ni*, no-imaging; *pt*, models with pre-training; *nd*, use of neuropsychology and demographic information.

Table A9: Prediction of conversion to dementia at two years.

| Model | MCC | AUC | F1-score |
|---|---|---|---|
| RF-ni | 0.409 | 0.666 | 0.452 |
| RF | 0.000 | 0.500 | 0.000 |
| FFN | 0.093 | 0.531 | 0.148 |
| CNN | -0.029 | 0.496 | 0.000 |
| GNN | 0.378 | 0.602 | 0.333 |
| FFN-pt | 0.378 | 0.602 | 0.333 |
| CNN-pt | 0.527 | 0.723 | 0.562 |
| GNN-pt | 0.587 | 0.730 | 0.600 |
| RF-nd | 0.376 | 0.579 | 0.273 |
| FFN-nd | 0.434 | 0.670 | 0.467 |
| CNN-nd | 0.434 | 0.670 | 0.467 |
| GNN-nd | 0.583 | 0.684 | 0.538 |
| FFN-pt-nd | 0.497 | 0.677 | 0.500 |
| CNN-pt-nd | 0.586 | 0.772 | 0.629 |
| GNN-pt-nd | 0.581 | 0.707 | 0.570 |

Abbreviations: MCC, Matthews correlation coefficient; AUC, area under the curve; RF, random forest; FFN, feed-forward network; CNN, convolutional neural network; GNN, graph neural network; *ni*, no-imaging; *pt*, models with pre-training; *nd*, use of neuropsychology and demographic information.

Table A10: Prediction of conversion to dementia at four years.

| Model | MCC | AUC | F1-score |
|---|---|---|---|
| RF-ni | 0.597 | 0.764 | 0.643 |
| RF | 0.627 | 0.744 | 0.640 |
| FFN | 0.517 | 0.788 | 0.595 |
| CNN | 0.331 | 0.588 | 0.300 |
| GNN | 0.466 | 0.651 | 0.455 |
| FFN-pt | 0.533 | 0.773 | 0.606 |
| CNN-pt | 0.721 | 0.878 | 0.765 |
| GNN-pt | 0.722 | 0.781 | 0.720 |
| RF-nd | 0.418 | 0.645 | 0.435 |
| FFN-nd | 0.613 | 0.790 | 0.667 |
| CNN-nd | 0.705 | 0.853 | 0.750 |
| GNN-nd | 0.597 | 0.764 | 0.643 |
| FFN-pt-nd | 0.677 | 0.847 | 0.727 |
| CNN-pt-nd | 0.764 | 0.909 | 0.800 |
| GNN-pt-nd | 0.691 | 0.827 | 0.733 |

Abbreviations: MCC, Matthews correlation coefficient; AUC, area under the curve; RF, random forest; FFN, feed-forward network; CNN, convolutional neural network; GNN, graph neural network; *ni*, no-imaging; *pt*, models with pre-training; *nd*, use of neuropsychology and demographic information.

Table A11: Prediction of diagnostic transition over a 4-year period.

| Model | MCC | AUC | F1-score |
|---|---|---|---|
| RF-ni | 0.308 | 0.670 | 0.448 |
| RF | 0.129 | 0.578 | 0.325 |
| FFN | 0.214 | 0.628 | 0.385 |
| CNN | 0.019 | 0.511 | 0.229 |
| GNN | 0.279 | 0.653 | 0.424 |
| FFN-pt | 0.191 | 0.601 | 0.349 |
| CNN-pt | 0.484 | 0.762 | 0.585 |
| GNN-pt | 0.406 | 0.720 | 0.523 |
| RF-nd | 0.166 | 0.582 | 0.316 |
| FFN-nd | 0.336 | 0.699 | 0.474 |
| CNN-nd | 0.280 | 0.647 | 0.419 |
| GNN-nd | 0.367 | 0.684 | 0.483 |
| FFN-pt-nd | 0.332 | 0.672 | 0.459 |
| CNN-pt-nd | 0.561 | 0.795 | 0.645 |
| GNN-pt-nd | 0.461 | 0.755 | 0.567 |

Abbreviations: MCC, Matthews correlation coefficient; AUC, area under the curve; RF, random forest; FFN, feed-forward network; CNN, convolutional neural network; GNN, graph neural network; *ni*, no-imaging; *pt*, models with pre-training; *nd*, use of neuropsychology and demographic information.

Table A12: Correlation between predicted and true values for continuous cognitive decline.

| Model | Memory | Language | Visuospatial | Executive |
|---|---|---|---|---|
| RF-ni | 0.25 [0.11, 0.38] | 0.45 [0.29, 0.58] | 0.54 [0.37, 0.67] | 0.35 [0.20, 0.48] |
| RF | 0.42 [0.27, 0.55] | 0.33 [0.16, 0.48] | 0.32 [0.12, 0.48] | 0.46 [0.32, 0.58] |
| FFN | 0.31 [0.16, 0.45] | 0.45 [0.30, 0.58] | 0.28 [0.13, 0.42] | 0.46 [0.30, 0.60] |
| CNN | 0.33 [0.18, 0.45] | 0.31 [0.14, 0.45] | 0.27 [0.09, 0.42] | 0.35 [0.20, 0.48] |
| GNN | 0.44 [0.29, 0.57] | 0.34 [0.17, 0.49] | 0.35 [0.19, 0.50] | 0.47 [0.32, 0.59] |
| FFN-pt | 0.46 [0.32, 0.59] | 0.47 [0.33, 0.60] | 0.32 [0.17, 0.46] | 0.48 [0.34, 0.62] |
| CNN-pt | 0.55 [0.41, 0.68] | 0.58 [0.44, 0.71] | 0.51 [0.35, 0.65] | 0.59 [0.46, 0.69] |
| **GNN-pt** | 0.53 [0.37, 0.66] | 0.57 [0.43, 0.70] | 0.48 [0.32, 0.63] | **0.61 [0.49, 0.73]** |
| RF-nd | 0.44 [0.29, 0.57] | 0.40 [0.24, 0.54] | 0.55 [0.39, 0.69] | 0.51 [0.36, 0.65] |
| FFN-nd | 0.39 [0.23, 0.53] | 0.41 [0.25, 0.54] | 0.35 [0.18, 0.50] | 0.38 [0.21, 0.52] |
| CNN-nd | 0.37 [0.21, 0.52] | 0.44 [0.27, 0.57] | 0.50 [0.35, 0.65] | 0.40 [0.25, 0.54] |
| **GNN-nd** | 0.43 [0.27, 0.57] | 0.45 [0.27, 0.59] | **0.56 [0.41, 0.69]** | 0.54 [0.38, 0.68] |
| FFN-pt-nd | 0.48 [0.33, 0.61] | 0.51 [0.35, 0.65] | 0.41 [0.25, 0.55] | 0.52 [0.36, 0.66] |
| **CNN-pt-nd** | **0.57 [0.44, 0.70]** | **0.60 [0.46, 0.71]** | 0.53 [0.38, 0.66] | 0.60 [0.47, 0.73] |
| GNN-pt-nd | 0.56 [0.40, 0.68] | 0.56 [0.41, 0.68] | 0.49 [0.33, 0.63] | 0.59 [0.46, 0.69] |

The 95% confidence intervals, presented in square brackets, were estimated from 1,000 bootstrap iterations on the test set. Abbreviations: *ni*, no-imaging; *pt*, models with pre-training; *nd*, use of neuropsychology and demographic information.

Table A13: Area under the ROC curve (AUC) for models predicting binary cognitive decline.

| Model | Memory | Language | Visuospatial | Executive |
|---|---|---|---|---|
| RF-ni | 0.72 [0.64, 0.80] | 0.72 [0.64, 0.80] | 0.63 [0.55, 0.71] | 0.71 [0.63, 0.79] |
| RF | 0.72 [0.62, 0.80] | 0.74 [0.66, 0.82] | 0.62 [0.53, 0.70] | 0.74 [0.66, 0.82] |
| FFN | 0.69 [0.58, 0.77] | 0.75 [0.68, 0.83] | 0.63 [0.55, 0.71] | 0.71 [0.62, 0.79] |
| CNN | 0.72 [0.63, 0.81] | 0.75 [0.67, 0.82] | 0.61 [0.52, 0.68] | 0.73 [0.66, 0.81] |
| GNN | 0.74 [0.66, 0.82] | 0.71 [0.62, 0.79] | 0.67 [0.60, 0.74] | 0.73 [0.65, 0.81] |
| FFN-pt | 0.76 [0.67, 0.83] | 0.79 [0.72, 0.86] | 0.68 [0.60, 0.76] | 0.77 [0.69, 0.83] |
| CNN-pt | 0.83 [0.77, 0.89] | 0.86 [0.80, 0.91] | 0.71 [0.64, 0.79] | 0.81 [0.74, 0.88] |
| GNN-pt | 0.81 [0.73, 0.88] | 0.85 [0.78, 0.90] | 0.68 [0.60, 0.75] | 0.81 [0.75, 0.88] |
| RF-nd | 0.73 [0.64, 0.81] | 0.76 [0.68, 0.83] | 0.63 [0.55, 0.71] | 0.74 [0.66, 0.82] |
| FFN-nd | 0.71 [0.63, 0.80] | 0.79 [0.72, 0.85] | 0.59 [0.51, 0.67] | 0.73 [0.65, 0.81] |
| CNN-nd | 0.72 [0.64, 0.80] | 0.74 [0.66, 0.81] | 0.63 [0.55, 0.72] | 0.72 [0.63, 0.79] |
| GNN-nd | 0.77 [0.69, 0.84] | 0.76 [0.68, 0.83] | 0.69 [0.61, 0.76] | 0.73 [0.64, 0.80] |
| FFN-pt-nd | 0.77 [0.69, 0.85] | 0.81 [0.74, 0.87] | 0.69 [0.62, 0.76] | 0.77 [0.69, 0.84] |
| **CNN-pt-nd** | 0.84 [0.78, 0.89] | **0.88 [0.83, 0.92]** | 0.71 [0.63, 0.78] | 0.82 [0.76, 0.88] |
| **GNN-pt-nd** | **0.84 [0.78, 0.89]** | 0.85 [0.79, 0.90] | **0.73 [0.66, 0.80]** | **0.83 [0.77, 0.90]** |

The 95% confidence intervals, presented in square brackets, were estimated from 1,000 bootstrap iterations on the test set. Abbreviations: *ni*, no-imaging; *pt*, models with pre-training; *nd*, use of neuropsychology and demographic information.

# References


[1] Edmund T Rolls, Chu-Chung Huang, Ching-Po Lin, Jianfeng Feng, and Marc Joliot. Automated anatomical labelling atlas 3. *Neuroimage*, 206:116189, 2020.

[2] Juergen Dukart, Karsten Mueller, Annette Horstmann, Barbara Vogt, Stefan Frisch, Henryk Barthel, Georg Becker, Harald E Möller, Arno Villringer, Osama Sabri, et al. Differential effects of global and cerebellar normalization on detection and differentiation of dementia in fdg-pet studies. *Neuroimage*, 49(2):1490–1495, 2010.

[3] Fei Tony Liu, Kai Ming Ting, and Zhi-Hua Zhou. Isolation forest. In *2008 eighth ieee international conference on data mining*, pages 413–422. IEEE, 2008.

[4] Fabian Pedregosa, Gaël Varoquaux, Alexandre Gramfort, Vincent Michel, Bertrand Thirion, Olivier Grisel, Mathieu Blondel, Peter Prettenhofer, Ron Weiss, Vincent Dubourg, et al. Scikit-learn: Machine learning in python. *the Journal of machine Learning research*, 12:2825–2830, 2011.

[5] Lijuan Ren, Tao Wang, Aicha Sekhari Seklouli, Haiqing Zhang, and Abdelaziz Bouras. A review on missing values for main challenges and methods. *Information Systems*, 119:102268, 2023.

[6] Joseph F. Hair, G. Tomas M. Hult, Christian M. Ringle, Marko Sarstedt, Nicholas P. Danks, and Soumya Ray. *An Introduction to Structural Equation Modeling*, pages 1–29. Springer International Publishing, Cham, 2021.
